# Development of a resource modelling tool to support decision makers in pandemic influenza preparedness: The AsiaFluCap Simulator

**DOI:** 10.1186/1471-2458-12-870

**Published:** 2012-10-12

**Authors:** Mart Lambertus Stein, James W Rudge, Richard Coker, Charlie van der Weijden, Ralf Krumkamp, Piya Hanvoravongchai, Irwin Chavez, Weerasak Putthasri, Bounlay Phommasack, Wiku Adisasmito, Sok Touch, Le Minh Sat, Yu-Chen Hsu, Mirjam Kretzschmar, Aura Timen

**Affiliations:** 1 National Institute for Public Health and the Environment, Centre for Infectious Disease Control, Bilthoven, 3720, BA, The Netherlands; 2 Utrecht Centre for Infection Dynamics, University Medical Centre Utrecht, Heidelberglaan 100, Utrecht, 3584, CX, Netherlands; 3 Communicable Disease Policy Research Group, London School of Hygiene and Tropical Medicine, Mahidol University, Satharanasukwisit Building, 420/1 Rajvithi Road, Bangkok, 10400, Thailand; 4 Municipal Health Service (GGD), Flevoland, Post box 1120, Lelystad, 8200 BC, The Netherlands; 5 Bernhard Nocht Institute for Tropical Medicine, Bernhard Nocht Str. 74, Hamburg, 20359, Germany; 6 Hamburg University of Applied Sciences, Lohbrügger Kirchstrasse 65, Hamburg, 21033, Germany; 7 Department of Preventive and Social Medicine, Faculty of Medicine Chulalongkorn University, 1873 Rama 4 Road, Pathumwan, Bangkok, 10330, Thailand; 8 Faculty of Tropical Medicine, Mahidol University, 420/6 Rajvithi Road, Bangkok, 10400, Thailand; 9 International Health Policy Program - Thailand, Ministry of Public Health, Tiwanond Road, Amphur Muang, Nonthaburi, 11000, Thailand; 10 National Emerging Infectious Diseases Coordination Office, Ministry of Health, Simoung, Sisatanak District, Vientiane, Lao PDR; 11 Faculty of Public Health, University of Indonesia, UI Campus, Depok, 16424, Indonesia; 12 Department of Communicable Disease Control, Ministry of Health, No. 151-153 Kampuchea Krom Blvd, Phnom Penh, Cambodia; 13 Ministry of Science and Technology of the Socialist Republic of Vietnam, 113 Tran Duy Hung street, Ha Noi, Vietnam; 14 Centers for Disease Control, R.O.C. (Taiwan), Taipei City, 10050, Taiwan R.O.C

**Keywords:** Pandemic influenza, Preparedness, Pandemic exercises, Public health officials, Decision making, Health care resources, Influenza modelling, Simulator

## Abstract

**Background:**

Health care planning for pandemic influenza is a challenging task which requires predictive models by which the impact of different response strategies can be evaluated. However, current preparedness plans and simulations exercises, as well as freely available simulation models previously made for policy makers, do not explicitly address the availability of health care resources or determine the impact of shortages on public health. Nevertheless, the feasibility of health systems to implement response measures or interventions described in plans and trained in exercises depends on the available resource capacity. As part of the AsiaFluCap project, we developed a comprehensive and flexible resource modelling tool to support public health officials in understanding and preparing for surges in resource demand during future pandemics.

**Results:**

The AsiaFluCap Simulator is a combination of a resource model containing 28 health care resources and an epidemiological model. The tool was built in MS Excel© and contains a user-friendly interface which allows users to select mild or severe pandemic scenarios, change resource parameters and run simulations for one or multiple regions. Besides epidemiological estimations, the simulator provides indications on resource gaps or surpluses, and the impact of shortages on public health for each selected region. It allows for a comparative analysis of the effects of resource availability and consequences of different strategies of resource use, which can provide guidance on resource prioritising and/or mobilisation. Simulation results are displayed in various tables and graphs, and can also be easily exported to GIS software to create maps for geographical analysis of the distribution of resources.

**Conclusions:**

The AsiaFluCap Simulator is freely available software (http://www.cdprg.org) which can be used by policy makers, policy advisors, donors and other stakeholders involved in preparedness for providing evidence based and illustrative information on health care resource capacities during future pandemics. The tool can inform both preparedness plans and simulation exercises and can help increase the general understanding of dynamics in resource capacities during a pandemic. The combination of a mathematical model with multiple resources and the linkage to GIS for creating maps makes the tool unique compared to other available software.

## Background

In the first decade of this century the world has faced three major crises due to infectious diseases: the Severe Acute Respiratory Syndrome (SARS) outbreak [1,2], the spread of the H5N1 Highly Pathogenic Avian Influenza virus (HPAI) [2,3] and the 2009 H1N1 Influenza pandemic [4].These events illustrate the continuous threat from emerging pathogens and underpin the need for a thorough preparedness and a robust response. According to the United Nations System Influenza Coordination (UNSIC)-World Bank fifth global progress report [2], the response to the 2009 pandemic revealed ‘substantial world-wide progress’ with pandemic preparedness since 2005. Most countries in Southeast Asia, a region where the H5N1 HPAI virus continues to circulate, have developed or updated their preparedness plans. In addition, national governments in this region have built considerable experience with simulation exercises [2,5]. However, such exercises and preparedness plans mostly do not take into account availability of health care resources, and therewith ignore the capacity of health systems to implement response measures that are described in plans and trained in exercises.

Outbreak response strategies involve, besides coordinated action by a number of stakeholders, the deployment of often limited health care resources (such as hospital facilities, antiviral drugs, hospital personnel, and personal protective equipment). During an outbreak, decision makers face surges in resource demand which require resource prioritisation and re-allocation, especially in economically constrained settings such as in low- and middle-income countries [6,7]. Concerns about the availability of critical care services during the first phase of the pandemic (H1N1) 2009 endorsed the importance of health care resource planning, even in the event of mild pandemics [8-10].

### Simulation exercises

A simulation exercise is a way to train users of a response plan and to evaluate the effectiveness of the plan. The purpose of these exercises is to enhance knowledge and understanding of the plan, and identify gaps, weaknesses and opportunities for improvement in planning and operational capabilities [11]. Most of the exercises performed in Southeast Asia were tabletop exercises where public health officials and/or key staff with emergency management responsibilities examine, discuss and manage a hypothetical pandemic situation in a round-table manner. For example, Thailand had at least one tabletop exercise at the central level and in each province. Lao PDR conducted tabletop exercises, in collaboration with the World Health Organization (WHO), with representatives from the Ministry of Health and other key players. There were also a few cross border tabletop exercises, coordinated by WHO and the Mekong Basin Disease Surveillance Network (MBDS).

In Indonesia, the first full-scale simulation exercise (of its kind in the world) was carried out, in which multiple ministries and agencies across central, provincial and district levels of the government participated. The exercise in Indonesia covered key areas for outbreak control, like surveillance (e.g. for early detection of human-to-human transmission), pharmaceutical (e.g. antiviral drugs and vaccines) and non-pharmaceutical interventions (e.g. social distancing measures), medical response (case management and isolation of cases) and risk communication (to the population and media) [5].

Most of the conducted simulation exercises focused on early containment (the early detection and control of outbreaks), but not on pandemic preparedness in later phases. There has been much less investment into preparing health systems for pandemic mitigation [12]. Nevertheless, the mitigation phase includes the peak period where the highest case number of infections is reached, and thus contains the peak demand for resources.

### Simulation tools

All preparedness plans and simulation exercises need a scenario with a hypothetical outbreak situation [11]. The development of a scenario requires a strong evidence base that provides the foundation for planning and response exercises [13]. In recent years it has become common to use mathematical modelling for guiding the response to disease outbreaks and support policy decisions [3,14-22]. Policy makers and other stakeholders involved in pandemic preparedness require (input from) flexible models to estimate and compare the effect of different intervention strategies during an influenza pandemic. Moreover, such mathematical tools should be easily adaptable for other outbreak scenarios to address the yearly differences in virus transmission and virulence, lack of understanding of factors affecting the spread of influenza and shortage of data on the effectiveness of interventions [23].

In the past, several simulation tools have been made publicly available [24-33]. These tools can be divided into applications that use a static modelling approach (e.g. FluSurge [26], FluAid [25] and StatFlu [24]) and those having a dynamic modelling structure (e.g. InfluSim [30], FluTE [28] and GLEaMviz [31]). All simulation tools can be used by policy makers to model a pandemic scenario in which an influenza virus is transmitted in a population and to estimate the burden of disease (e.g. the number of hospitalisations and deaths), conditional on given disease parameters. For example, StatFlu combines static modelling using historic influenza data with an easy-to-use interface and can be used to create insight in the effects of changing assumptions related to disease severity (like the attack rate and susceptibility of age groups) [24,31]. With the GLEaMviz Simulator users can configure a disease compartment model and a scenario to simulate, by setting compartment-specific variables, transitions, environmental effects and other conditions. In addition, with the GLEaMviz Simulator results can be explored in dynamics maps and charts that describe quantitatively the geotemporal distribution of the disease [31].

However, most models only address a few types of healthcare resources. For instance, Flusurge 2.0, which was developed by the US Centers for Disease Control and Prevention (CDC), is a tool that compares needed hospital resources during pandemic influenza with existing hospital resources, but only includes three items (hospital beds, intensive care beds, and mechanical ventilators) [34]. InfluSim, a tool that is flexible regarding several disease parameter values and with which the impact of multiple interventions can be estimated, includes only the available number of health care workers and antiviral drugs [30]. Moreover, all freely available simulation tools do not estimate operational resource capacity gaps nor their impact on public health during influenza pandemics.

For policy makers and advisors it is essential to have an indication on the regional distribution of health care resources, potential resource gaps and their impact on public health during a pandemic. Limited supplies force decision makers to determine how and when to deploy the available resources and to prioritise between resources [20,21]. Such decision-making requires models to provide estimates on surpluses and shortages of multiple outbreak related resources in geographical regions, during various pandemic scenarios. Understanding the dynamics of resources during a pandemic, including the consequences of resource gaps on public health and critical care services, can be of additional value to preparedness plans and simulation exercises such as tabletop exercises.

In summary, there is a need for a user-friendly and flexible resource modelling tool, which includes an easy-to-use option to display simulation results in maps, that could be used by policy makers and other stakeholders involved in pandemic preparedness for evidence based health care resource planning. Our objective was to develop such resource modelling tool, the AsiaFluCap Simulator, to support public health officials in understanding and preparing for surges in resource demand during future influenza pandemics. The development of this tool was part of a larger study, the AsiaFluCap project (http://www.cdprg.org), which aimed to provide a strategic framework to evaluate health system capacity in Cambodia, Indonesia, Lao PDR, Taiwan, Thailand, and Vietnam in response to different phases of pandemic influenza. The AsiaFluCap project was conducted from May 2008 to April 2011 with funding support from the European Union and the Rockefeller Foundation. The following sections outline the structure, applicability, benefits and limitations of the AsiaFluCap Simulator.

## Implementation

### Framework AsiaFluCap Simulator

The development of modelling tools involves a concession making process. It is a trade off between the usability and accessibility of the tool versus elements which may make the model more realistic (such as the use of individual based structures which can be used to estimate the geographical spread of infectious diseases [3,35,36]). As our main focus was to estimate the impact on health care resource capacity during influenza pandemics, we used a resource model in combination with a relatively simple epidemiological model (developed earlier in the AsiaFluCap project [12] and previously applied in resource gap analysis in countries in Southeast Asia [37,38]). Our other objectives were to produce an easy-to-use and easily accessible tool, allowing the use by policy makers and other health care professionals without modelling expertise.

**Figure 1:**
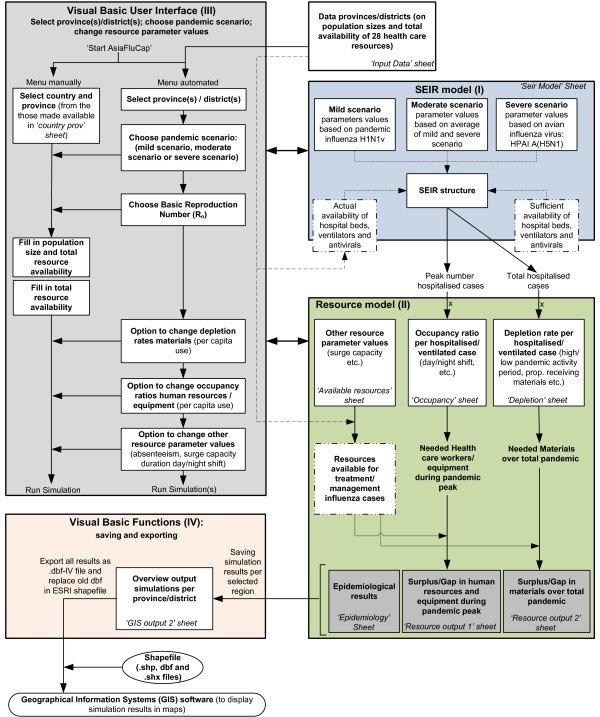
Schematic overview of the AsiaFluCap Simulator structure and processes.

The structure of the AsiaFluCap Simulator tool was based on a framework described by Eriksson et al. 2007 [39]. The tool consists of (I) an epidemiological model combined with (II) a resource model containing 28 health care resources, (III) a graphical user interface, and (IV) a function to export simulation results to GIS software for displaying simulation results in maps (Figure 1). Part I and II of the AsiaFluCap Simulator require, next to information on available resource capacities and population sizes in areas, influenza and resource parameter values. We developed the tool in Microsoft Excel© (Microsoft Corporation, Redmond, WA) and the user interface was developed using the programming language VBA (Visual Basic for Applications) in Visual Basic. The tool is compatible with all Microsoft Excel© versions (except Microsoft Excel Viewer©).

### Disease model

In order to estimate the resource demand during an influenza pandemic, one requires estimates of the total number of influenza cases, especially concerning the peak number of hospitalised cases and cases with specific treatment and care, e.g. cases requiring mechanical ventilation or intensive care units (ICUs). For this, we used a deterministic SEIR (Susceptible-Exposed-Infectious-Removed) model described by differential equations tracking number of people in each compartment over time. Full details of the epidemiological mode can be found in Krumkamp *et al.* 2011 [12], an additional file provides a detailed summary of the equations and assumptions [see Additional file 1. Given that the primary aim of our tool was to demonstrate relative differences in resource shortages and surpluses during different pandemic scenarios, rather then to provide accurate quantitative predictions, we used a relatively simple model structure assuming homogenously mixing and without an age-structure.

In the SEIR model the population is divided into 17 compartments, with the infectious compartment being subdivided into three groups based on clinical severity (asymptomatic, mild and severe infections). All severe cases were at risk of death, and assumed to need hospitalisation and antiviral treatment (of which a certain proportion also required mechanical ventilation). All asymptomatically and mild infected patients were assumed to recover. Hospitalisation and treatment with antivirals were assumed to reduce the infectious period and the probability of death for severe cases. We also assumed that a proportion of severe cases would require mechanical ventilation, without which they would die.

The SEIR model differs from other existing transmission models [26,30] as three key health care resources (hospital beds, mechanical ventilators, and antiviral drugs) were included as dynamic variables. Whether infected individuals received hospitalised care, ventilation or antiviral treatment depended on the availability of these resources. The inclusion of these resources as dynamic variables allows for quantitative estimates of the impact of resource shortages on morbidity and mortality.

#### Three pandemic influenza scenarios

The AsiaFluCap Simulator contains three pre-defined pandemic influenza scenarios: a mild pandemic scenario (based on Pandemic (H1N1) 2009 parameter values), a severe pandemic scenario (partly based on highly pathogenic avian influenza (HPAI) H5N1 parameter values, assuming a person-to-person transmission rate similar to human viruses) and a moderate pandemic scenario which is based on an average of the mild and severe disease parameter values. The underlying disease-specific parameters describing the transmissibility and clinical severity for these scenarios were based on data reported in the literature [8,40-45], additional tables provide the non-scenario and scenario specific parameter values used in the simulator [see Additional files 2 and 3. The three scenarios differ in parameter values regarding the proportion of mild and severe cases (i.e. cases requiring hospitalisation; proportion of severe cases requiring mechanical ventilation and the proportion of lethal cases.

#### Variable basic reproduction number

The tool allows for varying the basic reproduction number (R0), defined as the number of secondary infections produced by a single infectious individual in an otherwise susceptible population [46], between 1.2 and 2.5 for each included pandemic scenario. The R0 range was chosen based on a literature review [42]. With the given R0, the disease model estimates the transmissibility of the influenza virus (e.g. the proportion of contacts resulting in transmission), taking into account the infectious periods of the different case groups and the proportions asymptomatic and symptomatic cases.

#### Including interventions

The disease model contains the option to include non-pharmaceutical and pharmaceutical interventions, such as vaccination and antiviral treatment (although whether these interventions are implemented depends on the availability of the resources). Furthermore, it is possible to take into account contact reduction (e.g. due to social distancing and/or hygiene measures) in the general population or in hospitals, by providing the proportion with which the contact rate is reduced (during periods when over 0.5% of the population is symptomatically infected).

### Resource model

The epidemiological model estimations were explicitly linked to a resource model. The resource model consists of 28 health care resources with accompanying parameters reflecting the use of these resources per influenza case per (hospitalised) day. We distinguished between depleting and occupied resources. Depleting resources are items that can only be used once, like personal protective equipment (e.g. masks N-95/N-99, surgical masks, face shields, surgical gloves, and surgical coverall gowns), medications (e.g. antibiotics, antivirals, and IV fluids), vaccines, and body bags. For depleting resources it is essential to have an indication whether the capacity is sufficient for the entire pandemic outbreak and, in case of shortages, to have an idea on the moment of resource depletion.

Occupied resources are those which are occupied by influenza cases for a certain period, but can be redeployed such as human resources (e.g. medical doctors, nurses, general practitioners, internal medicine specialists, other types of doctors, pharmacists, laboratory technicians, public health personnel, volunteers, and administrative staff) and equipment (e.g. hospital beds, ICU, ambulances, other transport vehicles, x-ray machines, mechanical ventilators). For occupied resources the peak of the pandemic is the most critical point for maintaining services, as the number of cases then reaches its maximum.

To estimate the required quantities of resources (for each type of resource) during a pandemic, the output of the SEIR model (namely the estimated number of hospitalised and ventilated cases at the pandemic peak and during the total pandemic period) is multiplied in the resource model with the resource demands per capita (e.g. the number of resources needed per hospitalised case per day). Next, in order to calculate resource “surpluses” or shortages for the simulated pandemic scenario, the estimated required quantities are subtracted from the resource capacity available for outbreak control (which is a percentage of the total number of resources present in a region). To estimate resource surpluses and gaps, the resource model requires information on the resource demand per case and the total number of resources available in the region(s). In terms of resources, data on the available capacity of hospital beds, antiviral drugs, and mechanical ventilators are required in order to run the simulator (while the inclusion of other resource data is optional). An additional document provides a detailed overview of the resource model structure and assumptions [see Additional file 4].

The included resources in the tool were selected through a health system resource needs analyses which was also part of the AsiaFluCap project (http://www.cdprg.org). This resource characterisation process was carried out to inform policy makers in Southeast Asia on outbreak related resources and consisted of a systematic review and Delphi consensus (conducted in February 2009 by a panel of 24 public health experts from six countries in Southeast Asia and three countries from Europe).

#### Resource demand parameter values

The simulator contains depletion rates and occupancy ratios to reflect the use of resources per influenza case per day. For depleting resources, we included an optional function to change the use of resources per case over time. The rationale for including this function is that during a pandemic, hospitals will adjust their policy regarding resource use according to the number of cases requiring hospitalisation at a given time point. To account for these possible changes in resource use, we assumed that a pandemic is divided into two (hypothetical) periods, a low and high pandemic activity period. The AsiaFluCap Simulator splits the total pandemic period into two based on a threshold, which is determined by the proportion of hospital beds available for influenza cases that are not yet occupied by cases. For instance, if more than 75% of all hospital beds (available in the region for outbreak management) are occupied by influenza cases, then the model estimates resource needs by using the resource parameter values given for the high pandemic activity period. This threshold value is arbitrary and can be defined by the user in the tool.

The tool also contains other resource factors, like surge capacity percentages. These values indicate the percentages of the total number of general resources present in a region (which is provided by users) that could be made available for the management and control of influenza cases [47,48]. For the availability of human resources, we included mean number of work hours per week, duration of day and night shifts and absenteeism percentages. The AsiaFluCap Simulator contains by default resource parameter values which are based on a literature study by the authors, a previous resource study for Thailand [49] and interviews with public health professionals participating in the AsiaFluCap project. These resource parameter values can differ strongly between and even within countries, therefore all resource parameter values can be varied in the tool. An additional table displays all resource parameter values included in the tool [see Additional file 5.

#### Differences in resource needs calculations

The availability of hospital beds and ventilators limits the number of cases that can be hospitalised and ventilated, thus leading to severe outpatients when these resources are fully occupied. This interferes with resource needs calculations for all other included resources, as these estimations depend on the peak and total number of hospitalised cases. For instance, in case of insufficient hospital beds, the model underestimates the actual required quantities of hospital resources in case sufficient hospital beds would have been available. To prevent users from running each scenario twice for each region (e.g. one simulation with sufficient resources, another with actual available resources), the tool contains pre-simulated proportions for each possible scenario combination with sufficient resources (not assuming any interventions). The tool provides both the resource needs estimations made assuming sufficient hospital bed and ventilator capacity to accommodate all severe cases, and also the estimations assuming actual bed and ventilator capacity.

### Graphical user interface

In order to facilitate the use in practice we designed an interface in VBA for MS Excel© (Microsoft Corporation, Redmond, WA). The interface consists of six consecutive screens in which users are provided with technical background information about the AsiaFluCap Simulator and a menu in which different options for running simulations can be selected. An additional file displays a screenshot of the main menu of the interface [see Additional file 6]. The interface can be turned on by an extra button (‘Start AsiaFluCap’) that appears in the standard menu bar of MS Excel© (when the macro security settings of MS Excel© are set to ‘medium’ or ‘low’). The default values displayed in the interface are loaded from the individual sheets in the MS Excel© file.

Depending on whether users have provided data on the availability of resources in provinces or districts, users are provided with either a menu in which they need to fill in (manually) the available quantities in one region or a menu for selecting one or multiple provinces (automated menu). In case provinces or districts with corresponding data have been provided in the data input sheet, the regions are uploaded and displayed in the automated menu of the interface. Next, users can choose to simulate a mild, moderate or severe pandemic scenario with a specific R0. In three other provided menus it is possible to change all parameter values related to resource demands per case (including depletion rates in low and high pandemic activity periods, differences in resource demands between non-ventilated and ventilated hospitalised cases, and differences in case demands during day and night shifts) and change other resource parameter values (e.g. surge capacity percentages, mean number of work hours per week for healthcare personnel, duration of work shifts, and absenteeism percentages). All values provided in the interface are printed into the designated sheets in the tool for running simulations. The interface allows for resetting the default values from the literature (these default values are also loaded from the individual sheets). Additional files display screenshots of the menus for choosing pandemic scenarios, and changing and / or resetting depletion rates [see Additional files 7 and 8].

The interface contains help buttons for explaining functions and resource parameter values, and for displaying literature references of applied disease parameter values. The tool is also provided with an online audio-video guide in which the functions in the interface are explained (http://www.cdprg.org/asiaflucap-simulator.php). Running simulations with the AsiaFluCap Simulator takes around 15 minutes for 80 districts or provinces, depending on the available computing power. The use of the AsiaFluCap Simulator has been piloted during the AsiaFluCap project meeting in Lao PDR, with 15 policy makers and other health care professionals from six countries in Southeast Asia (Cambodia, Indonesia, Lao PDR, Taiwan, Thailand, and Vietnam). A second pilot was carried out in the Netherlands, in order to assess validity and feasibility for countries outside South-East Asia, and it involved the seven regional public health experts of the National Institute for Public Health and the Environment (RIVM) in the Netherlands.

### Saving and export function

We developed a VBA function that saves all simulation results each time per province or district, in a separate sheet in the simulator. With another VBA function these simulation results are exported, all or a selection of the results (only results related to hospital beds, mechanical ventilators, oseltamivir, physicians and nurses), in a .dbf-file (dBase IV format). A shapefile is a popular vector data format for Geographical Information Systems (GIS) software, for storing geometric location (in so-called ‘.shp’ and ‘.shx’ files) and associated information (in a ‘dbf-file’: attribute format) [50]. The simulator requires a shapefile, in which the dbf-file is automatically replaced (e.g. old file is renamed) with the new dbf-file from the tool, creating a ready-to-use shapefile containing all simulation results per province or district.

With (open-source) GIS software and the newly created shapefile, simulation results can be displayed in maps that illustrate the distribution of health care resources across districts or provinces in a country quantitatively. With GIS software it is possible to create maps of resource demands, gaps or surpluses and the impact of shortages on public health (e.g. number of deaths due to resource gaps per 100,000 population) across regions during a pandemic scenario, similar to those presented in previous resource gap analysis studies [37,38,49].

### Flexibility

The use of MS Excel© and the creation of the interface simplify the process of changing parameters in the model. The influenza scenarios in the model can be easily updated in the spreadsheets with values from future data on influenza or, if needed, changed to more extreme situations (e.g. a more mild of a more severe pandemic scenario). As data on the availability of 28 resources in regions or provinces might not be available to all policy makers, the AsiaFluCap Simulator also operates if only data on the availability of the three minimal needed resources (hospital beds, mechanical ventilators and antiviral drugs) is provided. Furthermore, other types of resources (with accompanying resource parameter values) can be added to the model (which will be automatically displayed in the interface) to also estimate the demand, shortage or surplus of these resources during a pandemic scenario.

## Sensitivity analysis

In the interface of tool it is possible to choose different pandemic scenarios with different basic reproduction numbers, and to change all resource parameter values to investigate the effects of changes in disease severity, virus transmissibility and resource demands per case on resource needs and shortages. Although not all disease parameter values can be varied in the interface, we provided the option for users (e.g. with modelling experience) to change all disease parameter values directly in the SEIR model sheet for an extensive sensitivity analysis.

## Simulation examples

To demonstrate the use of the AsiaFluCap Simulator for comparing different scenarios, we ran a mild scenario with the simulator for two provinces in Lao PDR (a low-income country in Southeast Asia [12]), namely Vientiane Prefecture (containing the capital Vientiane) and Vientiane Province. This region is located in the North-West of the country, and adjacent to Thailand, and has a total population size of around 1.1 million. Lao PDR, a country at high risk of being the epicentre of the next pandemic, is a member of the Mekong Basin Disease Surveillance (MBDS) initiative for cross-border surveillance and response [7]. To illustrate the influence of increased clinical severity on resource capacity, we also ran the simulator for a severe baseline scenario (no interventions and only severe cases are treated with antivirals). For both baseline scenarios we assumed a surge capacity of 12% (which is based on values described in earlier reports [47,48]), no interventions and only severe cases are treated with antivirals. We used a basic reproduction number of 1.4 (based on estimated reproduction numbers for pandemic (H1N1) 2009 derived for the North and North-East of Thailand, as found in [51]). We performed a sensitivity analysis with the tool by varying for all three pandemic scenarios the R0 between 1.2 and 2.5, to illustrate the effect of changes in virus transmissibility on the number of hospitalisations (assuming sufficient and actual available resources in the region).

To demonstrate the use of the AsiaFluCap Simulator for comparing resource gaps and surpluses between provinces for different pandemic scenarios, we also ran the simulator for every province in Lao PDR, and estimated required hospital beds, ventilators and oseltamivir during different pandemic situations. We ran simulations for all three pandemic baseline scenarios available in the tool (e.g. antiviral treatment only for severe cases and 12% surge capacity) and assuming a basic reproduction number of 1.4 [51]. Lao PDR has 17 provinces and a total population size of around 5.8 million [12]. Maps were created with ArcGIS version 10.

As part of the AsiaFluCap project, health system resource data were collected in Lao PDR (along with five other countries/territories: Cambodia, Indonesia, Taiwan, Thailand and Vietnam) between March and November 2009. Data on resource availability were collected through questionnaires administered to hospitals and health offices in all districts of each of the participating countries. Additional questionnaires were sent to ministries of health to capture central stockpiles [38].

## Results

### Resource estimations per region

**Figure 2:**
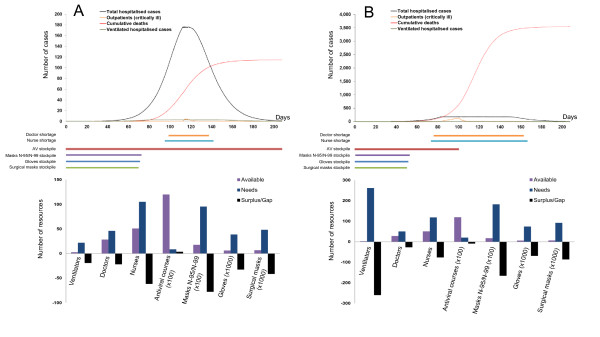
**Epidemiological results and impact on resource capacity during a mild and severe scenario.** Simulations made with the tool for a region in Lao PDR (Vientiane Prefecture and Vientiane Province, *n =* 1,099,889), using actual available resources. We assumed that 12% of the total resource capacity was available for treatment and care of pandemic cases. **A**: A mild baseline scenario. **B**: A severe baseline scenario for the same region. The bar charts directly below the graphs display the moment of depletion or, in case of occupied resources, the periods of shortages in hospital staff. Bar charts below display the available resources, and required quantities, gaps and surpluses.

The AsiaFluCap Simulator provides the simulation results in four different sheets in the tool. The model estimates the impact on public health, such as the number of hospitalised and ventilated cases and number of deaths, during a (selected) pandemic scenario. The tool reveals resource needs and, in case data on resource availability is provided, shortages and surpluses for the 28 included health care resources. It simulates the impact of limited resources (regarding hospital beds, ventilators and antivirals) on the outcome of the pandemic. Figure 2 displays graphs for the simulations made for the region in Lao PDR, as provided by the tool. A summary of the epidemiological estimations, assuming mild and severe pandemic scenarios in this region, is provided in Table 1. These key epidemiological estimations (e.g. total number of symptomatic cases stratified by disease subgroups, hospitalised subgroups and the cumulative number of deaths) and graphs with outbreak curves are provided in the ‘Epidemiology’ sheet of the tool. The next two sheets contain calculations on the impact on health care resource capacity, divided into depletion and occupied resources.

**Table 1:** Epidemiological estimations for a mild and a severe pandemic scenario

	Mild baseline scenario*	Severe baseline scenario*
Overall attack rate	421,704 (38.34%)	422,839 (38.44%)
Clinical attack rate	295,237 (26.84%)	297,295 (27.03%)
Peak prevalence of symptomatic cases	10,034 (0.91%)	10,017 (0.91%)
Peak prevalence hospitalised cases	176 (0.02%)	176 (0.02%)
Critical outpatients (over total pandemic)	11 (0.001%)	3018 (0.27%)
Case fatality rate	115 (0.01%)	3,543 (0.32%)

* Estimations made for a mild and severe baseline scenario for a region in Lao PDR (Vientiane Prefecture and Vientiane Province) assuming actual available resources, a basic reproduction number of 1.4 and 10% contact reduction. No other interventions were assumed and only severe cases were treated with antivirals. Values are provided in absolute numbers with the percentage of total population size (*n =* 1,099,889).

For resources that deplete, the tool calculates the required quantities, shortages or surpluses over the total pandemic period. These calculations are displayed in tables, bar charts and graphs, which provide a clear image of the resource output. In addition to the graphs that contain outbreak curves, the figures contain (for a number of resources) bars that provide an indication on the moment of depletion. For occupied resources, the AsiaFluCap Simulator displays the estimated resource needs, gaps or shortages, per pandemic peak day, in a table. The tables indicate resource gaps in red and surpluses in black. For medical doctors and nurses the tool provides insight on the moments of deficiencies in the number of available staff during the pandemic period.

The tool provides resource estimations both for a scenario for which sufficient available resources were assumed and a scenario with actual available resources. The difference between these two estimations is clearly illustrated in the simulations made for the region in Lao PDR. During a mild baseline scenario, hospital bed capacity in Lao PDR was (almost) sufficient enough to accommodate all severe cases (although there was a large shortage of ventilators). During a severe baseline scenario, this shortage in hospital beds limited the number of hospitalisations, leading to severe outpatients (Figure 2).

### Performing sensitivity analysis

The tool can be used to run different scenarios for the same region to compare the impact on resource needs during different pandemic outbreaks, but also to investigate the effect of changes in resource demands on disease burden. A summary of the sensitivity analysis performed with the tool for the region in Lao PDR is presented in an additional file [see Additional file 9]. Estimated resource needs are sensitive to the severity of the modelled pandemic scenario in terms of transmissibility (basic reproduction number) and the proportion of cases requiring hospitalisation. Furthermore, the peak numbers of hospitalisations, as well as the time of the peak, are sensitive to the chosen basic reproduction number.

### Simulations for multiple provinces

**Figure 3:**
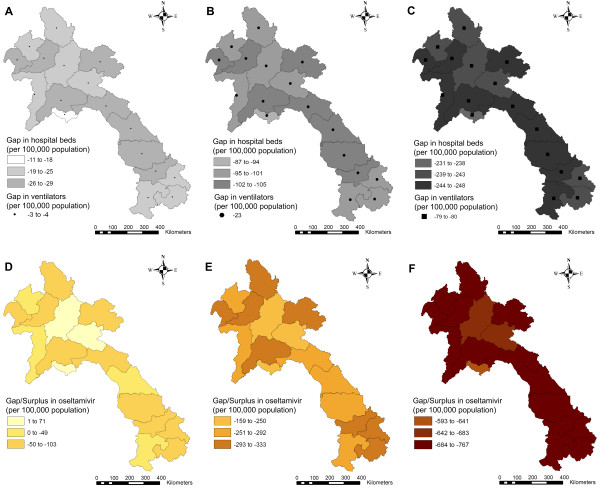
**Geographical distribution of estimated resource gaps across provinces in Lao PDR for three pandemic scenarios.** For demonstration purposes, the AsiaFluCap simulator was used to run for each province a mild (**A** and **D**), moderate (**B** and **E**) and severe (**C** and **F**) pandemic influenza scenario, assuming a basic reproduction number of 1.4 and contact reduction of 10%. The maps show surpluses and gaps in hospital beds and ventilators (**A**, **B**, and **C**), and oseltamivir (**D**, **E**, and **F**) for each pandemic scenario.

The AsiaFluCap Simulator runs a pandemic scenario for each province or district separately, taking into account the population size and the actual available resource capacity in regions. When the tool is used to run simulations for more than one province, then simulation results are provided per province in one overview table which is displayed in the fourth sheet of the tool. With the use of an added button in the menu bar of MS Excel©, the simulation results can be exported (partially or in total) and included in a shapefile (for displaying the simulator estimations per province or district in maps). Figure 3 displays the estimated gaps and surpluses in hospital beds, ventilators and oseltamivir, for each province in Lao PDR, during a mild, moderate and severe pandemic scenario. With these maps, provinces with (large) shortages or (abundant) surpluses in resources can be identified at a single glance, which could act as a trigger for policy makers for remobilisation or re-allocation of resources between (neighbouring) provinces. Although most provinces display insufficient resources for responding to the three modelled baseline scenarios, we did not demonstrated the effect of changes in resource parameter values (e.g. increased surge capacity) nor included interventions like social distancing measures. The AsiaFluCap Simulator can be easily used to compare other scenarios, next to the three baseline scenarios displayed in Figure 3.

## Discussion

By explicitly linking pandemic transmission dynamics to the usage of multiple healthcare resources, and also by facilitating the export of outputs to GIS software, the AsiaFluCap Simulator provides the user with additional benefits compared to existing pandemic prediction models. The user-friendly tool can be easily employed by policy makers, policy advisors, donors and other stakeholders involved in pandemic preparedness. The model can be used for providing evidence-based and illustrative information on health care system capacities during future pandemics. Such information can help inform preparedness and response plans and make participants of simulation exercises (like tabletop and ‘war room’ exercises) aware of surges in resource demand during pandemics. Furthermore, the tool could be applied for educational purposes, for example for learning the basics of mathematical modelling and understanding resource dynamics.

The flexibility of the tool allows policy makers to base scenario simulations upon assumptions currently made in national and regional strategic response plans to test the operationality of plans [40,49]. The tool can be used to provide insights on the potential impact of a pandemic on public health (e.g. estimations on the number of hospitalisations and deaths) and on health care resource capacity (e.g. estimations on required number of health care resources), indicate the geographical distribution of resource needs in a country (e.g. which districts or provinces are most likely to have resource shortages or surpluses?). Also, the availability of resources can be varied in simulations to explore the impact on public health of expanding health care resource capacity in regions. For instance, policy makers can explore whether increasing the number of hospital beds or mechanical ventilators may effectively reduce the number of deaths during a pandemic, which (provided there is sufficient data on resource effectiveness) could help guide decisions regarding resource prioritisation, potential investments or re-allocation of resources between regions.

In contrast to other pandemic models that include none or only a few resources [26,30], the AsiaFluCap Simulator concentrates primarily on health care resources that are likely to aid pandemic mitigation, particularly in terms of caring for severe cases. The tool does not currently include the possibility to directly display uncertainties in simulation estimates in graphs, as provided in previous resource gap analyses [38] and in the freely available model StatFlu [24]. Uncertainties in disease parameters can have a major impact on the epidemiological output with a strong effect on the underlying resource calculations [52]. Therefore, we provided different pandemic scenarios (with different values for clinical disease parameters) and the option to vary the transmissibility of influenza viruses in terms of the basic reproduction number. The remaining disease parameter values can be changed outside the interface. Meanwhile, all parameter values relating to resource usage can be changed in the interface of the tool.

Decision makers in pandemic preparedness often search for models providing the optimal combination of maximum realism, generality and precision at the same time [30]. Nevertheless, due to uncertainties in characteristics (e.g. the severity of disease) of future pandemics and in the effectiveness of various health care resources, there will be a continuing need to make decisions without definitive estimates [53,54]. Simple mathematical models, like the AsiaFluCap model, can be useful to explore a wide range of potential scenarios when only limited data are available [40,55]. The AsiaFluCap Simulator relies on relatively few basic parameters, allowing users to easily change both disease and resource parameter values if more recent data become available. The aim of the tool is therefore not to provide accurate information on exact quantities of resources needed, but to allow for comparative analysis of the effects of resource availability and the consequences of different strategies of resource use [56].

In the future, the AsiaFluCap Simulator could be extended with the incorporation of population heterogeneities such as high-risk groups, and age-groups with heterogeneous mixing patterns [56,57], as were included in the deterministic compartment model InfluSim [30]. These additional components will allow for exploration of the effects of various intervention strategies such as social distancing measures and vaccination of risk groups. Also, with an age-structured model resource needs could be differentiated per age group (e.g. paediatric and adult ventilators). As clearly shown in past influenza pandemics and in the pandemic (H1N1) 2009, the number of infected individuals, as well as hospitalisation rates are strongly age-dependent [2,44,58]. However, age-specific incidences and susceptibility to infection of future pandemics are not predictable. Moreover, an age-structured resource model would greatly increase data demands for the simulator, and age-stratified data on resource utilisation and effectiveness are currently scarce. It would also greatly increase computing time and requirements to run the simulator, and thus reduce its usability. Our main purpose was to provide a tool for policy makers that provides a first approximation to the problem of illustrating resource needs and impact of shortages during different pandemic scenarios.

The resource model could also be extended through inclusion of resource interdependencies. The management and control of influenza cases require more than one type of resources. For instance, hospitalised cases require at least a hospital bed, antiviral treatment and hospital personnel for care and treatment. A shortage in any one of these interdependent resources could affect the functioning of other resources. To account for this, more resources could be included in the epidemiological compartment model as dynamic variables, although parameterisation of such resource interdependencies would be challenging.

The concept of a resource simulator that estimates resource needs based on the output from simple or complex (e.g. individual-based) models could be applied to public health emergencies beyond pandemic influenza. Insights in health care resource needs and gaps during other events, like SARS or even natural disasters such as earthquakes, could provide useful support for policy makers facing these tremendous challenges. It is of vital importance to decision makers in disaster preparedness to have access to evidence-based information on effective ways to improve health-service response, especially for developing countries which often deal with both limited health care resources as well as with limited financial resources [7,20].

## Conclusions

The AsiaFluCap Simulator is a user-friendly, comprehensive and flexible simulation tool which can be used by decision makers involved in pandemic preparedness to estimate and compare the impact on health care resource capacity during different pandemic scenarios. The tool provides indications on resource gaps, impact of these gaps on public health and options for effectively improving resource capacity. The ease of exporting data to GIS software makes it possible to create illustrative maps for determining clusters of resource gaps and surpluses across districts and provinces. Such information can be used for national or regional plans and simulation exercises. Overall, the tool could help increase decision makers’ awareness and understanding of surges in resource demand during pandemics. The simulator is especially useful for developing countries where resources are limited and guidance on prioritisation and regional re-allocation is needed.

## Availability and requirements

The AsiaFluCap Simulator (beta version 2), accompanying manual, supporting materials and a video-audio guide can be freely downloaded from http://www.cdprg.org/asiaflucap-simulator.php. The Visual Basic source code for the interface is available upon request.

Project name: The AsiaFluCap project

Project home page: http://www.cdprg.org/asiaflucap.php

Operating system: MS Windows 2000 or newer

Programming language: MS Visual Basic

Other requirements: MS Excel©, (open-source) GIS software, 20 MB of hard drive storage space

License: none

## Competing interests

The authors have declared that they have no competing interests.

## Authors’ contributions

MLS wrote the first draft of the manuscript. JR, RC, CW, RK, AT contributed to writing of the manuscript. MLS, JR, CW, RK, MK, AT designed (the link between) the epidemiological and resource models. MLS, JR, RC, CW, RK, WP conducted literature reviews for model parameter values. WP, BP, WA, ST, LMS, YH played a key role in resource characterisation. RC, PH, BP coordinated data collection in Lao PDR. MLS, CW coded the simulator in MS Excel©. MLS designed the interface, conducted functional and system tests. CW, PH, IC, WP, BP, WA, ST, LMS, YH, MK contributed to the development of simulator functions. PH, IC contributed to the development of the link between the simulator and GIS software. RC, PH, IC, WP, BP, WA, ST, LMS, YH, AT contributed to piloting the simulator. AT led the model, scenario and interface development. RC led the AsiaFluCap project. All authors meet ICMJE criteria for authorship. All authors read and approved the final manuscript.

## Pre-publication history

The pre-publication history for this paper can be accessed here:

http://www.biomedcentral.com/1471-2458/12/870/prepub

## Supplementary Material

Additional file 1 **File format: Adobe Acrobat Document (.pdf) Title: Disease model Structure and Assumptions in the AsiaFluCap Simulator.** Description: Detailed description of the disease model structure and assumptions. Click here for file

Additional file 2 **File format: Adobe Acrobat Document (.pdf) Title: Table disease model parameters and values: non-scenario specific values.** Description: Table containing the non-scenario specific disease parameter values (with ranges and references) used in the AsiaFluCap Simulator. Click here for file

Additional file 3 **File format: Adobe Acrobat Document (.pdf) Title: Table disease model parameters and values: scenario specific.** Description: Table containing the disease parameter values (with ranges and references) for each of the three included pandemic scenarios in the AsiaFluCap Simulator. Click here for file

Additional file 4 **File format: Adobe Acrobat Document (.pdf) Title: Resource model structure and assumptions.** Description: Detailed description of the resource model and assumptions, explaining how resources were connected to the underlying disease model. Click here for file

Additional file 5 **File format: Adobe Acrobat Document (.pdf) Title: Resource model parameters and values.** Description: Table containing all resources model parameters and values (with references) used in the AsiaFluCap Simulator. Click here for file

Additional file 6 **File format: TIFF file (.tif) Title: Main menu of the AsiaFluCap Simulator interface.** Description: A screenshot of the main menu of the tool in which users can select provinces/districts (in screenshot are provinces in Thailand displayed), and enter the sub-menus of the interface. Click here for file

Additional file 7 **File format: TIFF file (.tif) Title: Screenshot of the pandemic scenario menu of the AsiaFluCap Simulator interface.** Description: A screenshot of the menu in which users can choose one of the three pandemic scenarios, change the basic reproduction number, and include contact reduction for their simulations. Click here for file

Additional file 8 **File format: TIFF file (.tif) Title: Screenshot of the depletion rate menu of the AsiaFluCap Simulator interface.** Description: A screenshot of the menu in which users can change depletion rates for different hospitalised cases (e.g. normal hospitalised and ventilated cases), and the proportions of cases receiving the resources. Buttons with a question mark explain definitions and assumptions related to resources. The reset button can be used to return to default values. Click here for file

Additional file 9 **File format: Adobe Acrobat Document (.pdf) Title: Sensitivity analysis performed with the AsiaFluCap Simulator.** Description: Simulations were done for a region in Lao PDR (Vientiane Prefecture and Vientiane Province, *n =* 1,099,889) for a mild, moderate and severe pandemic scenario, and assuming different values for R0 (1.2, 1.4, 1.6, 1.8, 2.0 and 2.5) and no interventions (e.g. contact reduction, vaccination, etc.). **A:** Number of hospitalised cases when assuming sufficient hospital beds available in the region. **B:** Number of hospitalised cases when running simulations with actual available resources in the region. **C:** Cumulative number of deaths due to resource gaps (calculated by subtracting the number of deaths during scenarios simulated at *A* from the number of deaths estimated from the scenarios simulated at *B*). Click here for file
