# Supplementary material for: Development of a resource modelling tool to support decision makers in pandemic influenza preparedness: The AsiaFluCap Simulator
Source: BMC Public Health. 2012 Oct 12;12:870. doi: 10.1186/1471-2458-12-870 (PMC3509032; doi:10.1186/1471-2458-12-870)
Supplement: Additional file 1 — File format: Adobe Acrobat Document (.pdf) Title: Disease model Structure and Assumptions in the AsiaFluCap Simulator. Description: Detailed description of the disease model structure and assumptions. [file 1471-2458-12-870-S1.pdf]

Additional file 1

Disease Model Structure and Assumptions

The AsiaFluCap Simulator

Introduction Disease Model AsiaFluCap Simulator

The AsiaFluCap Simulator is a combination of a disease model and a resource model. The resource model uses the pandemic scenario estimations from the disease model to calculate resource needs (see Additional file 4). As disease model, we adopted an existing SEIR model structure which was developed earlier, as part of the AsiaFluCap project, by Krumkamp and colleagues (2011) [1]. The disease model consists of a relatively simple SEIR structure assuming homogenously mixing patterns within a closed population. We chose to use a non-age-structured model as our main purpose of the AsiaFluCap Simulator was to demonstrate relative differences in resources needs during different pandemic scenarios, and not provide accurate quantitative predictions (e.g. development a tool for policy makers that provides a first approximation to the problem of illustrating the impact of limited resources on pandemic outcomes). Moreover, age-specific incidences and susceptibility to infection of future pandemics are not predictable, and an age-structured resource model would greatly increase data demands for the simulator. Such age-stratified data on resource utilisation and effectiveness are currently scarce.

The disease model parameters and values are described in separate tables (see Additional files 2 and 3). The resource model structure and assumptions are described in Additional file 4 and the table with resource parameters and values can be found in Additional file 5.

SEIR model structure

The disease model structure was based on a Susceptible-Exposed-Infectious-Removed (SEIR) model (fully detailed in Krumkamp *et al.* [1]. The population was divided into 17 compartments, defined as Susceptible (S); Exposed (E); Pre-symptomatic infectious cases (A); Asymptomatic infection (I_a and I_{a2}); Mild infection (I_m); Mild infection with antiviral treatment (I_{ma}), mild infection without antiviral treatment (I_{m2}); Critically ill requiring hospitalisation (I_c); Hospitalised and infectious (I_h); Hospitalised and non-infectious (I_{h2}); Critically ill at home with antiviral treatment (I_{ca}); Critically ill at home without antiviral treatment (I_{c2}); Ventilated and infectious (V); Ventilated and non-infectious (V_2); Dead (D); and Recovered (R). The differential equations of the model, defining the rates of change in these state variables over time, are displayed below:

$$\frac{dS}{dt} = -\Theta(\psi)\rho S - q\kappa(1-\alpha p_{\kappa})\frac{S}{N-D}(A + I_a + I_{a2} + I_m + I_{m2} + I_{ma} + I_c + I_{c2} + I_{ca}) - q(1-p_{qh})\kappa_h \frac{S}{N-D}(I_h + V) \quad (1)$$

$$\frac{dE}{dt} = q\kappa(1-\alpha p_{\kappa})\frac{S}{N-D}(A + I_a + I_{a2} + I_m + I_{m2} + I_{ma} + I_c + I_{c2} + I_{ca}) + q(1-p_{qh})\kappa_h \frac{S}{N-D}(I_h + V) - \sigma E \quad (2)$$

$$\frac{dA}{dt} = \sigma E - \delta A \quad (3)$$

$$\frac{dI_a}{dt} = \delta p_a A - \tau I_a \quad (4)$$

$$\frac{dI_{a2}}{dt} = \tau I_a - \gamma_a I_{a2} \quad (5)$$

$$\frac{dI_m}{dt} = \delta p_m A - \tau I_m \quad (6)$$

$$\frac{dI_{ma}}{dt} = \Theta(\varepsilon) p_{ma} \tau I_m - \gamma_{ma} I_{ma} \quad (7)$$

$$\frac{dI_{m2}}{dt} = \Theta(\varepsilon)(1-p_{ma})\tau I_m + (1-\Theta(\varepsilon))\tau I_m - \gamma_m I_{m2} \quad (8)$$

$$\frac{dI_c}{dt} = \delta p_c A - \tau I_c \quad (9)$$

$$\frac{dI_h}{dt} = \Theta(\varphi)(1-p_v)\tau I_c - \Theta(\varepsilon)\gamma_{ca} I_h - (1-\Theta(\varepsilon))\gamma_c I_h \quad (10)$$

$$\frac{dI_{h2}}{dt} = \Theta(\varepsilon)\gamma_{ca} I_h + (1-\Theta(\varepsilon))\gamma_c I_h - \gamma_h I_{h2} \quad (11)$$

$$\frac{dI_{ca}}{dt} = \Theta(\varepsilon) p_{ca}(1-\Theta(\varphi))(1-p_v)\tau I_c - \gamma_{ca} I_{ca} \quad (12)$$

$$\frac{dI_{c2}}{dt} = (1-\Theta(\varepsilon) p_{ca})(1-\Theta(\varphi))(1-p_v)\tau I_c - \gamma_c I_{c2} \quad (13)$$

$$\frac{dV}{dt} = \Theta(\omega) p_v \tau I_c - \Theta(\varepsilon)\gamma_{ca} V - (1-\Theta(\varepsilon))\gamma_c V \quad (14)$$

$$\frac{dV_2}{dt} = \Theta(\varepsilon)\gamma_{ca} V + (1-\Theta(\varepsilon))\gamma_c V - \gamma_v V_2 \quad (15)$$

$$\frac{dD}{dt} = \gamma_c d_c I_{c2} + \gamma_{ca} d_{ca} I_{ca} + \Theta(\varepsilon)\gamma_h d_{ha} I_{h2} + (1-\Theta(\varepsilon))\gamma_h d_h I_{h2} + \gamma_v d_v V_2 + (1-\Theta(\omega)) p_v \tau I_c \quad (16)$$

$$\frac{dR}{dt} = \Theta(\psi)\rho S + \gamma_a I_{a2} + \gamma_m I_{m2} + \gamma_{ma} I_{ma} + \gamma_c (1-d_c) I_{c2} + \gamma_{ca}(1-d_{ca}) I_{ca} + \Theta(\varepsilon)\gamma_h (1-d_{ha}) I_{h2} + (1-\Theta(\varepsilon))\gamma_h (1-d_h) I_{h2} + \gamma_v (1-d_v) V_2 \qquad (17)$$

Susceptible individuals (S) become infected at a force of infection, which depends on the per capita contact rate (k), the probability of transmission upon contact with an infectious person (q), and the proportion of individuals within the total population that are infectious. The AsiaFluCap Simulator contains the option for users to change the proportion with which the contact rate in the population is reduced, during periods when over 0.5% of the population is symptomatically infected. This was based on the assumption that during periods of increased pandemic activity, individuals will change their behaviour to reduce contacts, and/or social distancing measures such as school closure will be implemented.

Newly infected individuals move sequentially through an exposed (but not yet symptomatic or infectious) compartment (E), followed by an infectious, asymptomatic period (A). After this, a proportion (p_a) of those infected will remain asymptomatic, moving sequentially to compartments I_a and I_{a2}. Another proportion of infected individuals (p_m) will develop mild symptoms and move to compartment I_m, while the remaining proportion, p_c, will become critical cases with severe infections (I_c). We assumed that the asymptomatic, mild and critical case groups differ from each other in their duration of infectiousness. Only critical cases were at risk of dying from infection; all symptomatic and mild cases were assumed to recover.

We assumed that all critical cases require antiviral treatment and hospitalisation. A proportion of all hospitalised cases (p_v) requires mechanical ventilation, although whether they receive this depended upon the availability of antiviral drugs, hospital beds, and ventilators. These dynamic variables were defined by parameters ε, φ and ω respectively, which take a value of 1 if the resource is available, and 0 if the resource is depleted (in the case of antivirals) or fully occupied (in the case of beds and ventilators), see also Additional file 4.

If hospital beds are available, critical cases will become hospitalised and move to compartment I_h, otherwise they become critical outpatients with or without antiviral treatment (I_{ca} and I_{c2}, respectively), depending on the availability of antivirals. If ventilators are available, those cases requiring ventilation would move to the ventilated group (V_1), otherwise they are assumed to die. For these groups different death rates and different infectious periods apply according to the severity and whether they receive the healthcare resources they require. Treatment with antiviral drugs was assumed to reduce the infectious period and the probability of death. Hospitalised and ventilated cases were assumed to

remain in the hospital for a period after their infectiousness has disappeared, as denoted by compartments I_{h2} and I_{v2}, respectively.

For the baseline scenarios in the AsiaFluCap Simulator we assumed that only critical cases received antiviral treatment. However, if antivirals are available, a proportion of mild cases (p_{ma}) could also receive antiviral treatment and move to compartment I_{ma} (which is now set to zero in the simulator). Users can change the proportion of mild cases receiving antivirals in the interface, but also have the possibility to change the proportion of critical outpatients receiving antivirals (p_{ca}). Hospitalised and ventilated cases are assumed to always receive antivirals, when available. The AsiaFluCap Simulator only includes treatment with antivirals; not prophylactic use.

Users are also able to include vaccination in their scenario. Whether or not susceptibles are vaccinated depends on the availability of vaccines, which is defined with parameter ψ. Whether or not people get vaccinated at time *t* depends also on whether or not the targeted proportion of people being vaccinated has been reached (e.g. users can choose to vaccinate only a certain proportion of the total population). See for more information on vaccination Additional file 4.

Pandemic influenza scenarios AsiaFluCap Simulator

The AsiaFluCap Simulator contains three pandemic scenarios: a mild, moderate and severe scenario. The mild scenario was based on pandemic (H1N1) 2009 parameter values. The severe pandemic scenario was based on the highly pathogenic avian influenza (HPAI) H5N1 parameter values, assuming a person-to-person transmission rate similar to human viruses. The moderate scenario was defined by an average of the mild and severe parameter values. Distinction between these three scenarios consists of differences in the proportions of mild and critical cases; the proportion of critical cases requiring ventilation, and proportion of critical cases dying from influenza related illness.

For users with modelling experience the three pandemic scenarios can be easily changed (e.g. updated with the latest literature values) by unhiding the ‘SEIR model’ Excel sheet (In this sheet all disease parameter values are displayed) in the AsiaFluCap Simulator.

Variable Basic Reproduction Number (R_0)

The concept of R_0 is defined as the number of secondary infections produced by a single infectious individual in an otherwise susceptible population [2]. The R_0 is the most widely used epidemiological measurement of the transmission potential in a given population [3]. Besides the option in the AsiaFluCap Simulator to choose one of the three pandemic scenarios, users have the option to choose a R_0 (between 1.2 - 2.5, this range was chosen based on [4]) for their simulation run. With the selected R0, the disease model will estimate the transmissibility of the influenza virus (e.g. the proportion of

contacts resulting in transmission, q), taking into account the infectious periods of the different case groups and the proportions asymptomatic and symptomatic cases.

The variable R_0 and the pandemic scenarios can be used to compare the impact on resource needs and shortages on public health (impact of shortages only given for the dynamic variables hospital beds, ventilators and antivirals). By using the AsiaFluCap Simulator *interface*, 42 different scenarios (not including the use of interventions) can be selected for comparing the impact on resource capacity and public health.

References

1. Krumkamp R, Kretzschmar M, Rudge JW, Ahmad A, Hanvoravongchai P, Westenhoefer J, Stein M, Putthasri W, Coker R: **Health service resource needs for pandemic influenza in developing countries: a linked transmission dynamics, interventions and resource demand model**. *Epidemiol Infect* 2011:1-9.
2. Anderson R, May R: **Infectious Diseases of Humans**. Oxford, UK: Oxford University Press; 1992.
3. Nishiura H: **Correcting the actual reproduction number: a simple method to estimate R(0) from early epidemic growth data**. *International journal of environmental research and public health* 2010, **7**(1):291-302.
4. van der Weijden CP, Stein ML, Jacobi AJ, Kretzschmar MEE, Reintjes R, van Steenbergen JE, Timen A: **Choosing pandemic parameters for pandemic preparedness planning: A comparison of pandemic scenarios prior to and following the influenza A(H1N1) 2009 pandemic**. *Health Policy*, 2012.
