# Supplementary material for: Development of a resource modelling tool to support decision makers in pandemic influenza preparedness: The AsiaFluCap Simulator
Source: BMC Public Health. 2012 Oct 12;12:870. doi: 10.1186/1471-2458-12-870 (PMC3509032; doi:10.1186/1471-2458-12-870)
Supplement: Additional file 2 — File format: Adobe Acrobat Document (.pdf) Title: Table disease model parameters and values: non-scenario specific values. Description: Table containing the non-scenario specific disease parameter values (with ranges and references) used in the AsiaFluCap Simulator. [file 1471-2458-12-870-S2.pdf]

Additional file 2

Table Disease Model Parameters and Values: non-scenario specific values

The AsiaFluCap Simulator

**SEIR parameter**	**Description**	**Chosen value***	**Justification/notes**
κ	Rate of daily contacts possibly resulting in transmission	7	Assumption for the model.
σ	Rate at which individuals leave exposed group	1	1-2 days [1], 2.62 days [2], and 1-5 days [3] between infection and symptom onset.
δ	Rate at which individuals leave asymptomatic group	1/0.4	1 day [2, 3], and 0-2 days [3] infectious before symptom onset.
τ	Rate cases get detected	1/0.5	Assumption for the model.
γ_a	Rate at which asymptomatic cases leave infectious group	2	1.9 days [4], 3.38 days [2], 4-10 days [3] mean duration of infectivity.
γ_m	Rate at which mild cases leave infectious group	1/1.5	See above [2, 3, 5].
γ_c	Rate at which severe cases leave infected group	1/3.5	See above [2, 3, 5].
γ_{ma}<br>γ_{ca}	Rate at which mild and critical cases under AV treatment leave infectious groups	1/2.5	Infectious period under AV treatment reduced by 1 day [6].
γ_h	Rate at which cases leave hospital group	1/12	Length of ICU stay 13.5 days [5].
γ_v	Rate at which cases leave ventilated group	1/13	12 days [7], 15 days [8].
q	Proportion of contacts resulting in transmission	$q = R_0\gamma/\kappa$	Value estimated based on chosen R0 (which can be varied in interface between 1.2-2.5 [9]), taking into account contacts per day (κ) and different infectious periods of case groups (γ's) which are weighted according to the case proportions.
p_κ	Proportion of contacts reduced during contact reduction period (when prevalence of symptomatic cases >0.5%).	0.10	Value set to 0.10 for all baseline scenarios, which gives more realistic attack rates for given R0 values (since we have homogenous mixing). Users can change proportion in interface between 0 and 0.10.
p_{qh}	Proportion of hospital	0	Value set to 0 for baseline scenario. Users

	transmissions reduced (e.g. due to case isolation or use of PPE by hospital staff)		can change proportion in interface between 0 and 1.
p_{ma}	Proportion of mild cases treated with antivirals	0	Value set to 0 for baseline scenario. Users can change proportion in interface between 0 and 1.
p_{ca}	Proportion of critical outpatients treated with antivirals	1	For the baseline scenario it is assumed that only critical cases are treated with antivirals. Users can change proportion of critical outpatient receiving antivirals in interface between 0 and 1.
ρ	Rate at which susceptible individuals leave susceptible compartment through vaccination (number of individuals that are vaccinated per day).	0	Vaccination was not included in baseline scenario. Number of individuals that can be vaccinated per day can be changed by users in the interface.

*****All parameter values can be changed, either in interface or in the SEIR Model sheet of the AsiaFluCap Simulator.

References

1. Longini IM, Jr., Nizam A, Xu S, Ungchusak K, Hanshaoworakul W, Cummings DA, Halloran ME: **Containing pandemic influenza at the source**. *Science* 2005, **309**(5737):1083-1087.
2. Tuite AR, Greer AL, Whelan M, Winter AL, Lee B, Yan P, Wu J, Moghadas S, Buckeridge D, Pourbohloul B *et al*: **Estimated epidemiologic parameters and morbidity associated with pandemic H1N1 influenza**. *Cmaj* 2009, **182**(2):131-136.
3. Pourbohloul B, Ahued A, Davoudi B, Meza R, Meyers LA, Skowronski DM, Villasenor I, Galvan F, Cravioto P, Earn DJ *et al*: **Initial human transmission dynamics of the pandemic (H1N1) 2009 virus in North America**. *Influenza Other Respi Viruses* 2009, **3**(5):215-222.
4. Flahault A, Vergu E, Boelle PY: **Potential for a global dynamic of Influenza A (H1N1)**. *BMC Infect Dis* 2009, **9**:129.
5. Dominguez-Cherit G, Lapinsky SE, Macias AE, Pinto R, Espinosa-Perez L, de la Torre A, Poblano-Morales M, Baltazar-Torres JA, Bautista E, Martinez A *et al*: **Critically Ill patients with 2009 influenza A(H1N1) in Mexico**. *Jama* 2009, **302**(17):1880-1887.
6. Fraser C, Riley S, Anderson RM, Ferguson NM: **Factors that make an infectious disease outbreak controllable**. *Proc Natl Acad Sci U S A* 2004, **101**(16):6146-6151.
7. Kumar A, Zarychanski R, Pinto R, Cook DJ, Marshall J, Lacroix J, Stelfox T, Bagshaw S, Choong K, Lamontagne F *et al*: **Critically ill patients with 2009 influenza A(H1N1) infection in Canada**. *JAMA* 2009, **302**(17):1872-1879.

8. Jain S, Kamimoto L, Bramley AM, Schmitz AM, Benoit SR, Louie J, Sugerman DE, Druckenmiller JK, Ritger KA, Chugh R *et al*: **Hospitalized patients with 2009 H1N1 influenza in the United States, April-June 2009**. *N Engl J Med* 2009, **361**(20):1935-1944.
9. van der Weijden CP, Stein ML, Jacobi AJ, Kretzschmar MEE, Reintjes R, van Steenbergen JE, Timen A: **Choosing pandemic parameters for pandemic preparedness planning: A comparison of pandemic scenarios prior to and following the influenza A(H1N1) 2009 pandemic**. In: *Health Policy*. 2012.
