# Supplementary material for: Development of a resource modelling tool to support decision makers in pandemic influenza preparedness: The AsiaFluCap Simulator
Source: BMC Public Health. 2012 Oct 12;12:870. doi: 10.1186/1471-2458-12-870 (PMC3509032; doi:10.1186/1471-2458-12-870)
Supplement: Additional file 3 — File format: Adobe Acrobat Document (.pdf) Title: Table disease model parameters and values: scenario specific. Description: Table containing the disease parameter values (with ranges and references) for each of the three included pandemic scenarios in the AsiaFluCap Simulator. [file 1471-2458-12-870-S3.pdf]

Additional file 3

Table Disease Model Parameters and Values: scenario specific

The AsiaFluCap Simulator

SEIR parameter	Description	Scenario*	Value¶	Justification/notes
p_c	proportion of cases that are critical (require hospitalisation)	MiS	0.002	0.16% [1] and 0.45% [2] of symptomatic cases hospitalised.
		MoS	0.0065	Average of MiS and SeS
		SeS	0.015	CHR: 2.0% - 15.0% [3], 0.00345% - 3.36% depending on age groups [4], 6.0% [5], 0.8%-15.357% age-dependent [6], 5.0%- 13.0% [7].
p_a	Proportion of cases that are asymptomatic	MiS	0.30	Values range from 30 to 50% [2, 8, 9].
		MoS	0.30	
		SeS	0.30	
p_m	Proportion of cases that are mild	MiS	0.6980	pm=1-pa-pc
		MoS	0.6935	
		SeS	0.6850	
p_v	Proportion of hospitalised cases needing ventilation	MiS	0.1	Assumption based on: 10-39% of hospitalised patients admitted to ICU [10], 21% of hospitalised cases needing intensive care treatment [11], 18%-20% of hospitalised cases needing ventilation, 0.002-0.035% of symptomatic cases need ICU treatment [12-14].
		MoS	0.20	Average of MiS and SeS
		SeS	0.30	Assumption based on: 88% of H5N1 cases required ventilatory support for respiratory failure [15]. Respiratory failure is common, and some patients have developed the acute respiratory distress syndrome with very high mortality [16].
d_c	Proportion of critical outpatients without AV treatment that die	MiS	0.25	Proportion of deaths are extrapolated from the following assumptions: 41.4% (CI 28.9-55.0) CFR for critical illness [17], 17.3% (12.0-24.0) for critical illness [18], 11% of hospitalised cases died [14], 7% of hospitalised cases died [19], 0.007% of symptomatic

				cases died [1]. CFR 2.5% [20]. 0.00147-1.69% CFR depending on age groups [4].
		MoS	0.375	Average of MiS and SeS
		SeS	0.50	Proportion of deaths are extrapolated from the assumptions: Of the 573 H5N1 WHO-confirmed cases, 58.6% have resulted in death [21], CFR Human H5N1 cases ranged from 44% in Egypt to 80% in Indonesia [22, 23]. In 262 (61%) of sporadic cases of H5N1 virus infection reported (from 15 countries) the patient died [16]. 26 patients died out of 67 (CFR: 39%; 95% confidence interval, 27%–51%) [24]. The overall mortality rate of H5N1 cases was 56.5% [25].
d_{ca}	Proportion of critical outpatients under AV treatment that die	MiS	0.150	Based on $d_{ca} = d_c(1-\pi_a)$
		MoS	0.1875	
		SeS	0.200	
d_h	Proportion of hospitalised cases without AV treatment that die	MiS	0.100	Based on $d_h = d_c(1-\pi_h)$.
		MoS	0.178	
		SeS	0.280	
d_{ha}	Proportion of hospitalised cases under AV treatment that die	MiS	0.06	Based on $d_{ha} = d_c(1-\pi_a)(1-\pi_h)$.
		MoS	0.089	
		SeS	0.11	
d_v	Proportion of ventilated cases that die	MiS	0.25	Based on $d_v = (1- \pi_v)$.
		MoS	0.375	
		SeS	0.50	
π_a	Effectiveness of antiviral treatment at reducing death rate in critical cases	MiS	0.40	Assumption based on: [1, 17, 18]. Odds Ratio for reduction in influenza mortality in hospitalised cases: 0.21 (95% confidence interval 0.06-0.80) [26]. Patients who died were less likely to have received AV therapy within 48 hours after symptom onset [19].
		MoS	0.50	Average of MiS and SeS
		SeS	0.60	Assumption based on: of the 284 patients with known

				age and outcome who received either oseltamivir alone or no anti-influenza antiviral, the crude overall survival rate of patients who received at least 1 dose of oseltamivir alone (OS+) was 60%, whereas survival among AV− patients was 24% [25]. A higher proportion of cases survived that received any AV treatment compared to those that did not receive AV: 67% vs 7%, p = 0.003) [15].
π_h	Effectiveness of hospitalised care (without AV treatment) at reducing death rate in critical cases	MiS	0.6	Assumption based on [1, 17, 18], 7% CFR hospitalised cases [19].
		MoS	0.525	Average of MiS and SeS
		SeS	0.45	Assumption based on: 12% of hospitalised died within 1 day of admission, and 30% died within 2 days [27], 25.5% of hospitalised cased died 1918-19 pandemic [28]
π_v	Effectiveness of ventilators at preventing death in cases needing ventilation	MiS	0.75	Assumption based on [17, 18], 45% of ventilated cases died [19].
		MoS	0.625	Average of MiS and SeS
		SeS	0.50	Assumption based on: 12% of hospitalised died within 1 day of admission, and 30% died within 2 days [27]. Twenty-three (88%) of H5N1 cases required ventilatory support for respiratory failure, 65% of ventilated cases died [15]. Of the 7 who were ventilated, 2 survived [29].

***** MiS: Mild Scenario;
MoS: Moderate Scenario;
SeS: Severe Scenario
¶ All parameter values can be changed, either in interface or in the SEIR Model sheet.

References

1. Presanis AM, De Angelis D, Hagy A, Reed C, Riley S, Cooper BS, Finelli L, Biedrzycki P, Lipsitch M: **The severity of pandemic H1N1 influenza in the United States, from April to July 2009: a Bayesian analysis**. *PLoS Med* 2009, **6**(12):e1000207.

2. Reed C, Angulo FJ, Swerdlow DL, Lipsitch M, Meltzer MI, Jernigan D, Finelli L: **Estimates of the prevalence of pandemic (H1N1) 2009, United States, April-July 2009**. *Emerg Infect Dis* 2009, **15**(12):2004-2007.
3. Doyle A, Bonmarin I, Levy-Bruhl D, Le Strat Y, Desenclos JC: **Influenza pandemic preparedness in France: modelling the impact of interventions**. *Journal of epidemiology and community health* 2006, **60**(5):399-404.
4. Mylius SD, Hagenaars TJ, Lugner AK, Wallinga J: **Optimal allocation of pandemic influenza vaccine depends on age, risk and timing**. *Vaccine* 2008, **26**(29-30):3742-3749.
5. Wu JT, Riley S, Fraser C, Leung GM: **Reducing the impact of the next influenza pandemic using household-based public health interventions**. *PLoS Med* 2006, **3**(9):e361.
6. Haber MJ, Shay DK, Davis XM, Patel R, Jin X, Weintraub E, Orenstein E, Thompson WW: **Effectiveness of interventions to reduce contact rates during a simulated influenza pandemic**. *Emerg Infect Dis* 2007, **13**(4):581-589.
7. Carrat F, Luong J, Lao H, Salle AV, Lajaunie C, Wackernagel H: **A 'small-world-like' model for comparing interventions aimed at preventing and controlling influenza pandemics**. *BMC Med* 2006, **4**:26.
8. Longini IM, Jr., Nizam A, Xu S, Ungchusak K, Hanshaoworakul W, Cummings DA, Halloran ME: **Containing pandemic influenza at the source**. *Science* 2005, **309**(5737):1083-1087.
9. Duerr HP, Brockmann SO, Piechotowski I, Schwehm M, Eichner M: **Influenza pandemic intervention planning using InfluSim: pharmaceutical and non- pharmaceutical interventions**. *BMC Infect Dis* 2007, **7**:76.
10. World Health Organisation: **Transmission dynamics and impact of pandemic influenza A (H1N1) 2009 virus**. *Wkly Epidemiol Rec* 2009:481-484.
11. Siva N: **Number of swine flu patients going into intensive care is rising**. *BMJ* 2009, **339**:b4528.
12. Haghdoost AA, Gooya MM, Baneshi MR: **Modelling of H1N1 flu in Iran**. *Archives of Iranian medicine* 2009, **12**(6):533-541.
13. Ercole A, Taylor BL, Rhodes A, Menon DK: **Modelling the impact of an influenza A/H1N1 pandemic on critical care demand from early pathogenicity data: the case for sentinel reporting**. *Anaesthesia* 2009, **64**(9):937-941.
14. Louie JK, Acosta M, Winter K, Jean C, Gavali S, Schechter R, Vugia D, Harriman K, Matyas B, Glaser CA *et al*: **Factors associated with death or hospitalization due to pandemic 2009 influenza A(H1N1) infection in California**. *JAMA* 2009, **302**(17):1896-1902.
15. Yu H, Gao Z, Feng Z, Shu Y, Xiang N, Zhou L, Huai Y, Feng L, Peng Z, Li Z *et al*: **Clinical characteristics of 26 human cases of highly pathogenic avian influenza A (H5N1) virus infection in China**. *PLoS One* 2008, **3**(8):e2985.

16. Uyeki TM: **Human infection with highly pathogenic avian influenza A (H5N1) virus: review of clinical issues**. *Clin Infect Dis* 2009, **49**(2):279-290.
17. Dominguez-Cherit G, Lapinsky SE, Macias AE, Pinto R, Espinosa-Perez L, de la Torre A, Poblano-Morales M, Baltazar-Torres JA, Bautista E, Martinez A *et al*: **Critically Ill patients with 2009 influenza A(H1N1) in Mexico**. *Jama* 2009, **302**(17):1880-1887.
18. Kumar A, Zarychanski R, Pinto R, Cook DJ, Marshall J, Lacroix J, Stelfox T, Bagshaw S, Choong K, Lamontagne F *et al*: **Critically ill patients with 2009 influenza A(H1N1) infection in Canada**. *JAMA* 2009, **302**(17):1872-1879.
19. Jain S, Kamimoto L, Bramley AM, Schmitz AM, Benoit SR, Louie J, Sugerman DE, Druckenmiller JK, Ritger KA, Chugh R *et al*: **Hospitalized patients with 2009 H1N1 influenza in the United States, April-June 2009**. *N Engl J Med* 2009, **361**(20):1935-1944.
20. Flahault A, Vergu E, Boelle PY: **Potential for a global dynamic of Influenza A (H1N1)**. *BMC Infect Dis* 2009, **9**:129.
21. Wang TT, Parides MK, Palese P: **Seroevidence for H5N1 influenza infections in humans: meta-analysis**. *Science* 2012, **335**(6075):1463.
22. Organisation WH: **Epidemiology of WHO-confirmed human cases of avian influenza A(H5N1) infection**. *Wkly Epidemiol Rec* 2006, **81**(26):249-257.
23. Organisation WH: **Update: WHO-confirmed human cases of avian influenza A (H5N1) infection, November 2003-May 2008**. *Wkly Epidemiol Rec* 2008, **83**(46):415-420.
24. Liem NT, Tung CV, Hien ND, Hien TT, Chau NQ, Long HT, Hien NT, Mai le Q, Taylor WR, Wertheim H *et al*: **Clinical features of human influenza A (H5N1) infection in Vietnam: 2004-2006**. *Clin Infect Dis* 2009, **48**(12):1639-1646.
25. Adisasmito W, Chan PK, Lee N, Oner AF, Gasimov V, Aghayev F, Zaman M, Bamgboye E, Dogan N, Coker R *et al*: **Effectiveness of antiviral treatment in human influenza A(H5N1) infections: analysis of a Global Patient Registry**. *The Journal of infectious diseases* 2010, **202**(8):1154-1160.
26. McGeer A, Green KA, Plevneshi A, Shigayeva A, Siddiqi N, Raboud J, Low DE: **Antiviral therapy and outcomes of influenza requiring hospitalization in Ontario, Canada**. *Clin Infect Dis* 2007, **45**(12):1568-1575.
27. Kandun IN, Tresnaningsih E, Purba WH, Lee V, Samaan G, Harun S, Soni E, Septiawati C, Setiawati T, Sariwati E *et al*: **Factors associated with case fatality of human H5N1 virus infections in Indonesia: a case series**. *Lancet* 2008, **372**(9640):744-749.
28. Rizzo C, Ajelli M, Merler S, Pugliese A, Barbetta I, Salmaso S, Manfredi P: **Epidemiology and transmission dynamics of the 1918-19 pandemic influenza in Florence, Italy**. *Vaccine* 2011, **29 Suppl 2**:B27-32.
29. Le MT, Wertheim HF, Nguyen HD, Taylor W, Hoang PV, Vuong CD, Nguyen HL, Nguyen HH, Nguyen TQ, Nguyen TV *et al*: **Influenza A H5N1 clade 2.3.4 virus with a different**

antiviral susceptibility profile replaced clade 1 virus in humans in northern Vietnam. *PLoS One* 2008, **3**(10):e3339.
