# Supplementary material for: Development of a resource modelling tool to support decision makers in pandemic influenza preparedness: The AsiaFluCap Simulator
Source: BMC Public Health. 2012 Oct 12;12:870. doi: 10.1186/1471-2458-12-870 (PMC3509032; doi:10.1186/1471-2458-12-870)
Supplement: Additional file 4 — File format: Adobe Acrobat Document (.pdf) Title: Resource model structure and assumptions. Description: Detailed description of the resource model and assumptions, explaining how resources were connected to the underlying disease model. [file 1471-2458-12-870-S4.pdf]

Additional file 4
Resource Model Structure and Assumptions
The AsiaFluCap Simulator

Introduction Resource Model AsiaFluCap Simulator

The resource model of the AsiaFluCap Simulator uses the output of the SEIR model (see Additional file 1) to estimate the required number of resources needed during a pandemic scenario. In total, the AsiaFluCap Simulator contains 28 health care resources (categorised as human resources, equipment and materials). Three of these resources are processed into the SEIR model as dynamic variables, namely antivirals (ε), hospital beds (φ) and mechanical ventilators (ω). The required capacities of the other 25 health care resources are calculated by using either the total number of hospitalised/ventilated cases during the total pandemic or the peak number of hospitalised/ventilated cases, which are both estimated by the SEIR model. We distinguished between:

Depleting resources: Depleting resource can only be used once, like PPE, antibiotics and antivirals. For this type of resources it is essential to have an indication on whether the total available capacity is sufficient over the entire pandemic. We indicated the number of resource available at time *t* with $\mathbf{N}_{\mathrm{DepletingResourceavailable}}(\mathrm{t})$ (negative = shortage; positive = surplus).

Occupied resources: Occupied resources are resources which are occupied for a certain amount of time, but can be re-used again like human resources, hospital beds, ventilators and other equipment. For occupied resources the pandemic peak is the most critical point, as the number of hospitalised/ventilated cases then reaches its maximum. This is indicated with $\mathbf{N}_{OccupiedResourceAvailable}(\mathrm{t}_{peak})$: the number of resources available at the pandemic peak (negative value = shortage; positive = surplus). For these occupied health care resources we assumed that only a certain proportion of the total number of available resources would be available for pandemic influenza cases, which is indicated with $\mathbf{p}_{surge}$ (value can be changed by users in the interface). The remaining proportion is required for maintaining essential healthcare services.

As part of the AsiaFluCap project, health system resource data were collected in Cambodia, Lao PDR, Thailand (along with three other countries/territories: Indonesia, Taiwan, and Viet Nam) between March and November 2009. Data on total resource availability ($\boldsymbol{N}_{total}$) were collected through questionnaires administered to hospitals and health offices in all districts of each of the participating countries. Additional questionnaires were sent to ministries of health to capture central stockpiles.

Below we the described the resource model structure and assumptions. The resource parameter values can be found in a separate table (see Additional file 5).

1.0 Dynamic resource variables in SEIR model

The parameters *ε*, *φ* and *ω* in the SEIR model are conditional on the availability of antivirals, hospital beds and ventilators respectively during the pandemic (which take value 0 or 1 depending on the availability). This is defined as follows:

Availability of antiviral drugs (ε):
$$\Theta(\varepsilon)=\begin{cases}1, N_{AntiviralAvailable}(t)>0\\0, N_{AntiviralAvailable}(t)=0\end{cases}$$

Availability of hospital beds (φ):
$$\Theta(\phi)=\begin{cases}1, N_{TotalBedsAvailable}(t)>0\\0, N_{TotalBedsAvailable}(t)=0\end{cases}$$

Availability of medical ventilators (ω):
$$\Theta(\omega)=\begin{cases}1, N_{VentilatorsAvailable}(t)>0\xleftrightarrow{\text{AND}}N_{TotalBedsavailable}(t)>0\\0, N_{VentilatorsAvailable}(t)=0\xleftrightarrow{\text{OR}}N_{TotalBedsavailable}(t)=0\end{cases}$$

With the total number of hospital beds available at time *t* is defined as:

$$\mathbf{N}_{TotalBedsAvailable}(t) = (\mathbf{p}_{surge} * \mathbf{N}_{BedsTotal}) - (\mathbf{I}_h(t)+\mathbf{I}_{h2}(t) - (\mathbf{V}(t) + \mathbf{V}_2(t))$$

And the total number of ventilators (adult and paediatric) at time *t* defined as:

$$\mathbf{N}_{VentilatorsAvailable}(t) = (\mathbf{p}_{surge} * \mathbf{N}_{VentilatorsTotal}) - (\mathbf{V}(t) + \mathbf{V}_2(t))$$

Note that cases can only be ventilated is there are both ventilators and hospital beds available (e.g. the use of mechanical ventilators depends on the availability of hospital beds).

1.2 Limited and unlimited hospital beds/ventilators

Due to the dynamic variables hospital beds (*φ*) and ventilators (*ω*) in the SEIR model, running simulations with limited hospital beds/ventilators will lead to less critical influenza cases being hospitalised/ventilated (and more outpatients) than actually is needed. Therefore, for estimating the needed quantities for all other resources in the AsiaFluCap Simulator and to prevent having to run the AFC simulator twice, we pre-ran simulations for all three scenarios and all possible Basic Reproduction Numbers (1.2-2.5) with the AsiaFluCap Simulator with unlimited resources. The resulting proportions hospitalised and ventilated of these simulations can be found the ‘Proportions’ sheet of the AsiaFluCap Simulator.

The proportions ($\mathbf{I}_{hmaxprop}$, $\mathbf{I}_{h2maxprop}$, etc.) are used to estimate the needed number of human resources and equipment (occupied resources) during the pandemic peak. In the ‘Resource Output 2’ sheet of the simulator we provided the estimations made using these proportions. We also provided the estimated

number of human resources and equipment when actual available quantities of hospital beds and ventilators are used in the SEIR model (calculations made with $\mathbf{I}_{hmax}$, $\mathbf{I}_{h2max}$, etc).

Note that $\mathbf{I}_{hMaxprop} = \mathbf{I}_{hmax}$ if there are sufficient resources available in the target region.

2.0 Depleting resources

Depleting materials are materials that can only be used once, like antibiotics and antivirals. The needed numbers of materials are calculated over the total pandemic, using the total number of days cases are hospitalised (hospital case – days). Using days instead of number of cases is more properly for calculating resource needs, as the use of antiviral treatment for critical cases reduces the duration of hospital stay. In case you would calculate the needed number of resources with the total number of hospitalised cases you would overestimate the needed number resources as some cases are less long hospitalised (and therefore use less resources).

We used depletion rates (*Þ*) to indicate the use of resources per influenza case per day. We assumed that ventilated cases uses a number of times more resources per day than normal hospitalised cases, which is indicated with a factor, $\boldsymbol{F}_{Ventilatedresource}$ (if F is 1 then ventilated cases require the same amount of resources per day as normal hospitalised cases).

2.1 Use of threshold for depleting resources

We assumed that during the pandemic outbreak, hospitals adjust their policy regarding resource use, either by using less or more resources per case per day. To account for possible changes in resource use during the pandemic, the AsiaFluCap Simulator contains a ‘threshold’ ($\boldsymbol{p}_{threshold}$) which fictitiously divides the total pandemic period into a low and high pandemic activity period. This threshold is determined by the proportion of total hospital beds (available for pandemic influenza cases) that are occupied by cases at certain (unknown) point during the pandemic. By setting a threshold value in the AsiaFluCap Simulator, which can be chosen by users in interface, it can be indicate how many hospital beds have to be occupied by cases before the high pandemic activity period starts.

The simulator contains different depletion rates for the low pandemic activity period (($Þ_{ResourceLOW}$) and the high pandemic activity period ($Þ_{ResourceHIGH}$), to account for changes in resource use during the pandemic. For our baseline scenarios, we did not assume different depletion rates for the low and high pandemic activity.

The implementation of this threshold in the simulator can be defined as:

- Estimated number of hospital case – days corresponding to non-ventilated hospitalised cases below threshold at time *t*:

$$N_{NormalhospDaysBelow}(t) = \begin{cases} I_h(t) + I_{h2}(t), N_{BedsAvailable}(t) > p_{threshold} * (p_{surge} * N_{bedsTotal}) \\ 0, N_{BedsAvailable}(t) \leq p_{threshold} * (p_{surge} * N_{bedsTotal}) \end{cases}$$

- Estimated number of hospital case – days corresponding to non-ventilated hospitalised cases above threshold at time *t*:

$$N_{NormalhospDaysAbove}(t) = \begin{cases} I_h(t) + I_{h2}(t), N_{BedsAvailable}(t) \leq p_{threshold} * (p_{surge} * N_{bedsTotal}) \\ 0, N_{BedsAvailable}(t) > p_{threshold} * (p_{surge} * N_{bedsTotal}) \end{cases}$$

- Estimated number of hospital case – days corresponding to ventilated hospitalised cases below threshold at time *t*:

$$N_{VentilatedDaysBelow}(t) = \begin{cases} V(t) + V_2(t), N_{BedsAvailable}(t) > p_{threshold} * (p_{surge} * N_{bedsTotal}) \\ 0, N_{BedsAvailable}(t) \leq p_{threshold} * (p_{surge} * N_{bedsTotal}) \end{cases}$$

- Estimated number of hospital case – days corresponding to ventilated hospitalised cases above threshold at time *t*:

$$N_{VentilatedDaysAbove}(t) = \begin{cases} V(t) + V_2(t), N_{BedsAvailable}(t) \leq p_{threshold} * (p_{surge} * N_{bedsTotal}) \\ 0, N_{BedsAvailable}(t) > p_{threshold} * (p_{surge} * N_{bedsTotal}) \end{cases}$$

2.2 Estimation of specific materials

Below we defined how we estimated the number of materials available at time *t* in the AsiaFluCap model, taking into account the different depletion rates for the low ($\text{Þ}_{ResourceHIGH}$) and high ($\text{Þ}_{ResourceHIGH}$) pandemic activity periods and for the non-ventilated/normal hospitalised cases ($_{Normalhosp}$) and ventilated cases ($_{Ventilated}$).

2.2.1 Masks N-95/N-99 (PPE)

The estimated number of masks N-95/N-99 available at time *t*:

$$\mathbf{N}_{MaskN95Available}(t) = \mathbf{N}_{MaskN95Total} - \sum_{t=0}^{t} \big((\mathbf{N}_{NormalhospDaysBelow}(t) * \mathbf{Þ}_{maskn95LOW}) + (\mathbf{N}_{NormalhospDaysAbove}(t) * \mathbf{Þ}_{maskn95HIGH}) + (\mathbf{N}_{VentilatedDaysBelow}(t) * (\mathbf{F}_{ventilatedmask95} * \mathbf{Þ}_{maskn95LOW})) + (\mathbf{N}_{VentilatedDaysAbove}(t) * (\mathbf{F}_{ventilatedmask95} * \mathbf{Þ}_{maskn95HIGH})) \big)$$

2.2.2 Surgical masks (PPE)

The estimated number of surgical masks available at time *t*:

$$
\begin{aligned}
\mathbf{N}_{SurgicalmaskAvailable}(t) = \mathbf{N}_{SurgicalmaskTotal} - \sum_{t=0}^{t} \Big(& (\mathbf{N}_{NormalhospDaysBelow}(t) * \textbf{Þ}_{SurgicalmaskLOW}) \\
& + (\mathbf{N}_{NormalhospDaysAbove}(t) * \textbf{Þ}_{SurgicalmaskHIGH}) \\
& + (\mathbf{N}_{VentilatedDaysBelow}(t) * (\mathbf{F}_{ventilatedsurgmask} * \textbf{Þ}_{SurgicalmaskLOW}) \\
& + (\mathbf{N}_{VentilatedDaysAbove}(t) * \mathbf{F}_{ventilatedsurgmask} * \textbf{Þ}_{SurgicalmaskHIGH})) \Big)
\end{aligned}
$$

2.2.3 Face shields (PPE)

The estimated number of face shields available at time *t*:

$$
\begin{aligned}
\mathbf{N}_{FaceshieldAvailable}(t) = \mathbf{N}_{FaceshieldTotal} - \sum_{t=0}^{t} \Big(& (\mathbf{N}_{NormalhospDaysBelow}(t) * \textbf{Þ}_{FaceshieldLOW}) \\
& + (\mathbf{N}_{NormalhospDaysAbove}(t) * \textbf{Þ}_{FaceshieldHIGH}) \\
& + (\mathbf{N}_{VentilatedDaysBelow}(t) * (\mathbf{F}_{ventilatedfaceshield} * \textbf{Þ}_{FaceshieldLOW})) \\
& + (\mathbf{N}_{VentilatedDaysAbove}(t) * (\mathbf{F}_{ventilatedfaceshield} * \textbf{Þ}_{FaceshieldHIGH})) \Big)
\end{aligned}
$$

2.2.4 Gloves, pairs (PPE)

The estimated number of gloves (pairs) available at time *t*:

$$
\begin{aligned}
\mathbf{N}_{GlovespairAvailable}(t) = \mathbf{N}_{GlovespairTotal} - \sum_{t=0}^{t} \Big(& (\mathbf{N}_{NormalhospDaysBelow}(t) * \textbf{Þ}_{GlovespairLOW}) \\
& + (\mathbf{N}_{NormalhospDaysAbove}(t) * \textbf{Þ}_{GlovespairHIGH}) \\
& + (\mathbf{N}_{VentilatedDaysBelow}(t) * (\mathbf{F}_{ventilatedgloves} * \textbf{Þ}_{GlovespairLOW})) \\
& + (\mathbf{N}_{VentilatedDaysAbove}(t) * (\mathbf{F}_{ventilatedgloves} * \textbf{Þ}_{GlovespairHIGH})) \Big)
\end{aligned}
$$

2.2.5 Coverall Gowns (PPE)

The estimated number of gowns available at time *t*:

$$
\begin{aligned}
\mathbf{N}_{GownsAvailable}(t) = \mathbf{N}_{GownsTotal} - \sum_{t=0}^{t} \Big(& (\mathbf{N}_{NormalhospDaysBelow}(t) * \textbf{Þ}_{GownsLOW}) \\
& + (\mathbf{N}_{NormalhospDaysAbove}(t) * \textbf{Þ}_{GownsHIGH}) \\
& + (\mathbf{N}_{VentilatedDaysBelow}(t) * (\mathbf{F}_{ventilatedgowns} * \textbf{Þ}_{GownsLOW})) \\
& + (\mathbf{N}_{VentilatedDaysAbove}(t) * (\mathbf{F}_{ventilatedgowns} * \textbf{Þ}_{GownsHIGH})) \Big)
\end{aligned}
$$

2.2.6 Amoxicillin, grams (antibiotic)

We assumed that only certain proportions of non-ventilated/normal hospitalised cases ($\mathbf{p}_{NonventilatedAmoxicillin}$) and ventilated cases ($\mathbf{p}_{VentilatedAmoxicillin}$) require amoxicillin during their hospital stay. The estimated amoxicillin (in grams) available at time *t* can be defined as:

$$\mathbf{N}_{AmoxicillinAvailable}(t) = \mathbf{N}_{AmoxicillinTotal} - \mathbf{p}_{NonventilatedAmoxicillin} * \sum_{t=0}^{t} ((\mathbf{N}_{NormalhospDaysBelow}(t) * \mathbf{Þ}_{AmoxicillinLOW}) + (\mathbf{N}_{NormalhospDaysAbove}(t) * \mathbf{Þ}_{AmoxicillinHIGH})) - \mathbf{p}_{VentilatedAmoxicillin} * \sum_{t=0}^{t} ((\mathbf{N}_{VentilatedDaysBelow}(t) * (\mathbf{F}_{ventilatedAmoxicillin} * \mathbf{Þ}_{AmoxicillinLOW})) + (\mathbf{N}_{VentilatedDaysAbove}(t) * (\mathbf{F}_{ventilatedAmoxicillin} * \mathbf{Þ}_{AmoxicillinHIGH}))$$

2.2.7 Cotrimoxazole, grams (antibiotic)

We assumed that only certain proportions of non-ventilated/normal hospitalised cases ($\mathbf{p}_{NonventilatedCotrimoxazole}$) and ventilated cases ($\mathbf{p}_{VentilatedCotrimoxazole}$) require cotrimoxazole during their hospital stay. The estimated cotrimoxazole (in grams) available at time *t* can be defined as:

$$\mathbf{N}_{CotrimoxazoleAvailable}(t) = \mathbf{N}_{CotrimoxazoleTotal} - \mathbf{p}_{NonventilatedCotrimoxazole} * \sum_{t=0}^{t} ((\mathbf{N}_{NormalhospDaysBelow}(t) * \mathbf{Þ}_{CotrimoxazoleLOW}) + (\mathbf{N}_{NormalhospDaysAbove}(t) * \mathbf{Þ}_{CotrimoxazoleHIGH})) - \mathbf{p}_{VentilatedCotrimoxazole} * \sum_{t=0}^{t} ((\mathbf{N}_{VentilatedDaysBelow}(t) * (\mathbf{F}_{ventilatedCotrimoxazole} * \mathbf{Þ}_{CotrimoxazoleLOW})) + (\mathbf{N}_{VentilatedDaysAbove}(t) * (\mathbf{F}_{ventilatedCotrimoxazole} * \mathbf{Þ}_{CotrimoxazoleHIGH}))$$

2.2.8 IV Fluids: 0.9% Normal Saline Solution (liters)

We assumed that only certain proportions of non-ventilated/normal hospitalised cases ($\mathbf{p}_{NonventilatedIVfluids}$) and ventilated cases ($\mathbf{p}_{VentilatedIVfluids}$) require IV fluids during their hospital stay. The estimated IV fluids (in liters) available at time *t* can be defined as:

$$\mathbf{N}_{IVfluidsAvailable}(t) = \mathbf{N}_{IVfluidsTotal} - \mathbf{p}_{NonventilatedIVfluids} * \sum_{t=0}^{t} ((\mathbf{N}_{NormalhospDaysBelow}(t) * \mathbf{Þ}_{IVfluidsLOW}) + (\mathbf{N}_{NormalhospDaysAbove}(t) * \mathbf{Þ}_{IVfluidsHIGH}))$$

$$-$$

$$\mathbf{p}_{VentilatedIVfluids} * \sum_{t=0}^{t} ((\mathbf{N}_{VentilatedDaysBelow}(t) *(\mathbf{F}_{ventilatedIVfluids} * \mathbf{Þ}_{IVfluidsLOW})) + (\mathbf{N}_{VentilatedDaysAbove}(t) * (\mathbf{F}_{ventilatedIVfluids} * \mathbf{Þ}_{IVfluidsHIGH})))$$

2.2.9 Antiviral courses (oseltamivir)

In the simulator is assumed all hospitalised (including ventilated) cases receive antivirals, in case of sufficient availability. For our baseline scenarios we assumed that also all critical outpatients ($\boldsymbol{p}_{ca}$) receive antivirals (in case available). The AsiaFluCap Simulator allows for running scenarios where also a proportion of mild cases ($\boldsymbol{p}_{ma}$) receive antivirals. The estimated number of antiviral courses available at time *t* can be defined as:

$$\mathbf{N}_{AntiviralsAvailable}(t) = \mathbf{N}_{AntiviralsTotal} \sum_{t=0}^{t} - ((\mathbf{I}_{ma}(t) + \mathbf{I}_{ca}(t) + \mathbf{I}_{h}(t)+\mathbf{I}_{h2}(t)+\mathbf{V}(t)+\mathbf{V}_{2}(t)) * \mathbf{Þ}_{antivirals})$$

2.2.10 Vaccination (doses)

We assumed no vaccination strategies in our baseline scenarios.

However, the AsiaFluCap Simulator contains the option to include vaccination in simulations. In the interface of the tool users can indicate which proportion of the total population ($\boldsymbol{p}_{vac}$) they want to vaccinate effectively (which depends on vaccine efficacy $\boldsymbol{v}$). This can be defined as:

$$\mathbf{N}_{TargetVacEffective} = \boldsymbol{p}_{vac} * (1/\boldsymbol{v}) * \mathbf{S}(t_0)$$

and number of individuals vaccinated at time *t* as:

$$\mathbf{N}_{Vaccinated}(t) \quad \sum_{t=0}^{t} \Theta(\psi) \quad = \boldsymbol{\rho} * t$$ (see Additional file 1 for SEIR model)

The number of vaccines available at time t can be defined as:

$$\mathbf{N}_{VaccinesAvailable}(t) = (\mathbf{N}_{VaccinesTotal} / \mathbf{N}_{vaccinperperson}) - \sum_{t=0}^{t} \Theta(\psi)\, \boldsymbol{\rho} * t$$

with: $$\Theta(\psi) = \begin{cases} 1, N_{VaccinesAvailable}(t) > 0 \xleftrightarrow{and} N_{Vaccinated}(t) < N_{TargetVacEffective} \\ 0, N_{VaccinesAvailable}(t) = 0 \xleftrightarrow[or]{} N_{Vaccinated}(t) \geq N_{TargetVacEffective} \end{cases}$$

3.0 Occupied Resources

Occupied resources are health care resources that are occupied by cases, but become available again after a certain period, like human resources and equipment (hospital beds, ventilators, etc.). The pandemic peak (t_{peak}) is the most critical point during the pandemic, as the number of cases requiring hospitalisation/ventilation then reaches its maximum (defined as I_{hmax}, $I_{h2max,}$ V_{max} and V_{2max}).

For human resources, we assumed that healthcare workforce was divided into two day shifts (defined in hours: $\boldsymbol{H}_{hoursdayshiftTotal}$) and one night shift ($\boldsymbol{H}_{hoursnightshift}$). With ratios ($\boldsymbol{R}$) we indicated the number of cases one healthcare worker can treat or manage. Regarding the availability of healthcare workers, we assumed an average number of work hours per day seen over one week, which was calculated as work hours per day divided by number of working days per week ($\mathbf{H}_{averageworkhoursperday}$). We assumed that a certain proportion of health workers would not be available during the pandemic peak period due to absenteeism ($\mathbf{p}_{AbsenteeismPeak}$).

Note that for the calculations below we used both I_{hmax} (estimates from simulations using actual available resources) and $I_{hmaxprop}$ (with proportions from simulations with sufficient resources), see also 1.2.

3.1 Medical doctors / physicians

The estimated number of medical doctors/physicians available at the pandemic peak (t_{peak}):

$$\mathbf{N}_{MDphysiciansAvailable}(t_{peak}) = ((\mathbf{p}_{surge} * \mathbf{N}_{MDphysiciansTotal}) * (1 - \mathbf{p}_{AbsenteeismPeak})) - \Big((\mathbf{I}_{\mathrm{hmax}} + \mathbf{I}_{\mathrm{h2max}}) * ((\mathbf{H}_{hoursdayshiftsTotal} / 24h) * \mathbf{R}_{DayNonVentMDphysicians}) + ((\mathbf{H}_{hoursnightshift} / 24h) * \mathbf{R}_{NightNonVentMDphysicians})) + (\mathbf{V}_{\mathrm{max}} + \mathbf{V}_{\mathrm{2max}}) * ((\mathbf{H}_{hoursdayshiftsTotal} / 24h) * \mathbf{R}_{DayVentMDphysicians}) + (\mathbf{H}_{hoursnightshift} / 24h) * \mathbf{R}_{NightVentMDphysicians}) * (24 / \mathbf{H}_{averageworkhoursperday}) \Big)$$

3.2 General Practitioners / Primary Care Physicians

We assumed that a certain proportion of all critical outpatients and mild cases required a GP / Primary Care Physician ($\mathbf{p}_{casesvisitingGP}$). The estimated numbers available at the pandemic peak (t_{peak}) was calculated using the duration of a consultation (in hours: $\mathbf{H}_{hoursGPneededpercase}$), and can further be defined as:

$$\mathbf{N}_{GPsAvailable}(t_{peak}) = ((\mathbf{p}_{surge} * \mathbf{N}_{GPsTotal}) * (1 - \mathbf{p}_{AbsenteeismPeak})) - \Big(((\mathbf{I}_{\mathrm{maMax}} + \mathbf{I}_{\mathrm{maMax}} + \mathbf{I}_{\mathrm{caMax}} + \mathbf{I}_{\mathrm{caMax}}) * \mathbf{p}_{casesvisitingGP} * \mathbf{H}_{hoursGPneededpercase}) / \mathbf{H}_{averageworkhoursperday} \Big)$$

3.3 Internal medicine specialist / infectious disease specialists

The estimated number of internal medicine specialist / infectious disease specialists available at the pandemic peak (t_{peak}) can be defined as:

$$\mathbf{N}_{InternalMSAvailable}(t_{peak}) = ((\mathbf{p}_{surge} * \mathbf{N}_{InternalMSTotal}) * (1 - \mathbf{p}_{AbsenteeismPeak})) - \Big((\mathbf{I}_{\mathrm{hmax}} + \mathbf{I}_{\mathrm{h2max}}) * ((\mathbf{H}_{hoursdayshiftsTotal} / 24h) * \mathbf{R}_{DayNonVentInternalMS}) + ((\mathbf{H}_{hoursnightshift} / 24h) * \mathbf{R}_{NightNonVentInternalMS})) + (\mathbf{V}_{\mathrm{max}} + \mathbf{V}_{\mathrm{2max}}) * ((\mathbf{H}_{hoursdayshiftsTotal} / 24h) * \mathbf{R}_{DayVentInternalMS}) + (\mathbf{H}_{hoursnightshift} / 24h) * \mathbf{R}_{NightVentInternalMS}) * (24 / \mathbf{H}_{averageworkhoursperday}) \Big)$$

3.4 Other doctors (e.g. surgeons, pediatricians, obstetricians, etc.)

The estimated number of other doctors available at the pandemic peak (t_{peak}) can be defined as:

$$\mathbf{N}_{OtherDoctorsAvailable}(t_{peak}) = ((\mathbf{p}_{surge} * \mathbf{N}_{OtherDoctorsTotal}) * (1 - \mathbf{p}_{AbsenteeismPeak})) -$$

$$\Big((\mathbf{I}_{hmax} + \mathbf{I}_{h2max}) * ((\mathbf{H}_{hoursdayshiftsTotal} / 24h) * \mathbf{R}_{DayNonVentOtherDoctors}) + ((\mathbf{H}_{hoursnightshift} / 24h) * \mathbf{R}_{NightNonVentOtherDoctors})) + (\mathbf{V}_{max} + \mathbf{V}_{2max}) * ((\mathbf{H}_{hoursdayshiftsTotal} / 24h) * \mathbf{R}_{DayVentOtherDoctors}) + (\mathbf{H}_{hoursnightshift} / 24h) * \mathbf{R}_{NightVentOtherDoctors}) * (24 / \mathbf{H}_{averageworkhoursperday}) \Big)$$

3.5 Nurses

The estimated number of nurses available at the pandemic peak (t_{peak}) can be defined as:

$$\mathbf{N}_{NursesAvailable}(t_{peak}) = ((\mathbf{p}_{surge} * \mathbf{N}_{NursesTotal}) * (1 - \mathbf{p}_{AbsenteeismPeak})) -$$

$$\Big((\mathbf{I}_{hmax} + \mathbf{I}_{h2max}) * ((\mathbf{H}_{hoursdayshiftsTotal} / 24h) * \mathbf{R}_{DayNonVentNurses}) + ((\mathbf{H}_{hoursnightshift} / 24h) * \mathbf{R}_{NightNonVentNurses})) + (\mathbf{V}_{max} + \mathbf{V}_{2max}) * ((\mathbf{H}_{hoursdayshiftsTotal} / 24h) * \mathbf{R}_{DayVentNurses}) + (\mathbf{H}_{hoursnightshift} / 24h) * \mathbf{R}_{NightVentNurses}) * (24 / \mathbf{H}_{averageworkhoursperday}) \Big)$$

3.6 Pharmacists

We estimated the needed number of pharmacist by using the proportions ($_{MaxProp}$) of cases receiving antivirals and time needed for a pharmacist to prepare an antiviral course ($\mathbf{H}_{pharmacistpercase}$). Due to the structure of the SEIR model, it is not possible to estimate the number of cases receiving antivirals when running simulations with actual available resources (as all hospitalised / ventilated cases are assumed in the model to receive antivirals, when available). The estimated number of pharmacists available at the pandemic peak (t_{peak}) can be defined as:

$$\mathbf{N}_{pharmacistAvailable}(t_{peak}) = ((\mathbf{p}_{surge} * \mathbf{N}_{\mathrm{pharmacist}Total}) * (1 - \mathbf{p}_{AbsenteeismPeak})) - \left(((\mathbf{I}_{maMaxProp}(t) + \mathbf{I}_{caMaxProp}(t) + \mathbf{I}_{hMaxProp}(t) + \mathbf{I}_{h2MaxProp}(t) + \mathbf{V}_{MaxProp}(t) + \mathbf{V}_{2MaxProp}(t)) * \mathbf{H}_{pharmacistpercase}) / \mathbf{H}_{averageworkhoursperday} \right)$$

3.7 Laboratory technicians

We assumed that laboratory technicians only worked during day shifts. Regarding the availability of laboratory technicians we assumed a standard average number of work hours per day seen over one week, namely 7.1 hours (e.g. 5/7 work days; 10h work per day). And during this standard work day, we assumed that laboratory technicians can test a certain number of cases (indicated with $\mathbf{R}_{LaboratoryTCases}$). In the simulator the $\mathbf{H}_{averageworkhoursperday}$ can be changed (e.g. to investigate the effect in case laboratory technicians work longer). Only hospitalised and ventilated cases were assumed to be laboratory tested for influenza typing during the pandemic peak. The estimated number of laboratory technicians available at the pandemic peak (t_{peak}) can be defined as:

$$\mathbf{N}_{LaboratoryTAvailable}(t_{peak}) = ((\mathbf{p}_{surge} * \mathbf{N}_{LaboratoryTTotal}) * (1 - \mathbf{p}_{AbsenteeismPeak})) - \left((\mathbf{I}_{hmax} + \mathbf{I}_{h2max} + \mathbf{V}_{max} + \mathbf{V}_{2max}) * \mathbf{R}_{LaboratoryTCases} * (7.1h / \mathbf{H}_{averageworkhoursperday}) \right)$$

3.8 Public health personnel

We assumed that public health workers only worked during day shifts. Regarding the availability of public health workers we assumed a standard average number of work hours per day seen over one week, namely 7.1 hours (e.g. 5/7 work days; 10h work per day). We calculated the needed number of public health workers based on the number of cases during the pandemic peak. During a standard work day, we assumed that public health workers can manage a certain number of influenza cases (indicated with $\mathbf{R}_{PublicHealthPCases}$). The estimated number of public health workers available at the pandemic peak (t_{peak}) can be defined as:

$$\mathbf{N}_{PublicHealthPAvailable}(t_{peak}) = ((\mathbf{p}_{surge} * \mathbf{N}_{PublicHealthPTotal}) * (1 - \mathbf{p}_{AbsenteeismPeak})) - \left(((\mathbf{I}_{\mathrm{ma}} + \mathbf{I}_{\mathrm{m2}} + \mathbf{I}_{\mathrm{ca}} + \mathbf{I}_{\mathrm{c2}} + \mathbf{I}_{hmax} + \mathbf{I}_{h2max} + \mathbf{V}_{max} + \mathbf{V}_{2max}) * \mathbf{R}_{PublicHealthPCases}) * (7.1h / \mathbf{H}_{averageworkhoursperday}) \right)$$

3.9 Volunteers /community health workers

We assumed that volunteers / community health workers only worked during day shifts. Regarding the availability of volunteers /community health workers we assumed a standard average number of work hours per day seen over one week, namely 7.1 hours (e.g. 5/7 work days; 10h work per day). We calculated the needed numbers based on the number of cases during the pandemic peak. During a standard work day, we assumed that volunteers / community health workers can manage a certain number of influenza cases (indicated with $\mathbf{R}_{VolunteersCases}$). The estimated number of volunteers / community health workers available at the pandemic peak (t_{peak}) can be defined as:

$$\mathbf{N}_{VolunteersAvailable}(t_{peak}) = ((\mathbf{p}_{surge} * \mathbf{N}_{VolunteersTotal}) * (1 - \mathbf{p}_{AbsenteeismPeak})) - \Big((\mathbf{I}_{\mathrm{ma}} + \mathbf{I}_{\mathrm{m2}} + \mathbf{I}_{\mathrm{ca}} + \mathbf{I}_{\mathrm{c2}}\ \mathbf{I}_{hmax} + \mathbf{I}_{h2max} + \mathbf{V}_{max} + \mathbf{V}_{2max}) * \mathbf{R}_{VolunteersCases} * (7.1\mathrm{h} / \mathbf{H}_{averageworkhoursperday}) \Big)$$

3.10 Administrative staff hospital

We assumed that administrative staff only worked during day shifts. Regarding the availability of administrative staff we assumed a standard average number of work hours per day seen over one week, namely 7.1 hours (e.g. 5/7 work days; 10h work per day). During a standard work day, we assumed that for a certain number of cases one administrative worker is needed ($\mathbf{R}_{AdminStaffCases}$). The estimated number of administrative staff available at the pandemic peak (t_{peak}) can be defined as:

$$\mathbf{N}_{AdminStaffAvailable}(t_{peak}) = ((\mathbf{p}_{surge} * \mathbf{N}_{AdminStaffTotal}) * (1 - \mathbf{p}_{AbsenteeismPeak})) - \Big((\mathbf{I}_{hmax} + \mathbf{I}_{h2max} + \mathbf{V}_{max} + \mathbf{V}_{2max}) * \mathbf{R}_{AdminStaffCases} * (7.1\mathrm{h} / \mathbf{H}_{averageworkhoursperday}) \Big)$$

3.11 Standard hospital beds

We calculated the needed number of standard hospital beds (for non-ventilated) using the proportions ($_{Maxprop}$). We assumed that standard hospital beds can replace ICU beds in case there is a shortage of ICU beds. The estimated number of standard hospital beds available at the pandemic peak (t_{peak}) can be defined as:

$$\mathbf{N}_{StandardBedsAvailable}(t) = (\mathbf{p}_{surge} * \mathbf{N}_{standardBedsTotal}) - (\mathbf{I}_{hMaxProp}(t) - \mathbf{I}_{h2MaxProp}(t)$$

3.12 ICU beds (without mechanical ventilator)

We assumed that ICU was needed for ventilated cases. For the simulator, we assumed that ICU beds are not standard equipped with a mechanical ventilator. For all our scenarios we assumed that ICU beds could be supplemented with standard hospital beds. The estimated number of ICU beds available at the pandemic peak (t_{peak}) can be defined as:

$$\mathbf{N}_{ICUBedsAvailable}\ (t) = (\mathbf{p}_{surge} * \mathbf{N}_{ICUBedsTotal}) - \mathbf{V}_{MaxProp}(t) - \mathbf{V}_{2MaxProp}(t)$$

3.13 Ambulances

We assumed that a proportion of all hospitalised cases require an ambulance for transport to the hospital or between hospitals ($\mathbf{p}_{casesambulances}$). We took into account the duration of transport of one influenza case ($\mathbf{H}_{\mathrm{AmbulanceCase}}$), and assumed that an ambulance would 24 hours per day available. The estimated number of ambulances available at the pandemic peak (t_{peak}) can be defined as:

$$\mathbf{N}_{AmbulancesAvailable}(t_{peak}) = ((\mathbf{p}_{surge} * \mathbf{N}_{AmbulancesTotal}) - \Big(((\mathbf{I}_{hmax} + \mathbf{I}_{h2max} + \mathbf{V}_{max} + \mathbf{V}_{2max}) * \mathbf{H}_{\mathrm{AmbulanceCase}} * \mathbf{p}_{casesambulances}) / 24h \Big)$$

3.14 Other transport vehicles

We assumed that a proportion of all hospitalised cases require other vehicles (than ambulances) for transport to the hospital or between hospitals ($\mathbf{p}_{casesOthertransport}$). We took into account the duration of transport of one influenza case ($\mathbf{H}_{Othertransport\mathrm{Case}}$), and assumed that these vehicles would 24 hours per day available. The estimated number of other transport vehicles available at the pandemic peak (t_{peak}) can be defined as:

$$\mathbf{N}_{OthertransportAvailable}(t_{peak}) = ((\mathbf{p}_{surge} * \mathbf{N}_{OthertransportTotal}) - \Big(((\mathbf{I}_{hmax} + \mathbf{I}_{h2max} + \mathbf{V}_{max} + \mathbf{V}_{2max}) * \mathbf{H}_{Othertransport\mathrm{Case}} * \mathbf{p}_{casesOthertransport}) / 24h \Big)$$

3.15 X-ray/radiographic machines

We assumed that only a proportion of all ventilated cases require an X-ray ($\mathbf{p}_{Xray\mathrm{Case}}$). We took into account the duration of one x-ray / scan session ($\mathbf{H}_{Xray\mathrm{Case}}$). The estimated number of x-ray / radiographic machines available at the pandemic peak (t_{peak}) can be defined as:

$$\mathbf{N}_{XraysAvailable}(t_{peak}) = ((\mathbf{p}_{surge} * \mathbf{N}_{XraysTotal}) - \Big((\mathbf{V}_{max} + \mathbf{V}_{2max}) * \mathbf{H}_{Xray\mathrm{Case}} * \mathbf{p}_{Xray\mathrm{Case}}) / 24h \Big)$$
