# Supplementary material for: Development of a resource modelling tool to support decision makers in pandemic influenza preparedness: The AsiaFluCap Simulator
Source: BMC Public Health. 2012 Oct 12;12:870. doi: 10.1186/1471-2458-12-870 (PMC3509032; doi:10.1186/1471-2458-12-870)
Supplement: Additional file 5 — File format: Adobe Acrobat Document (.pdf) Title: Resource model parameters and values. Description: Table containing all resources model parameters and values (with references) used in the AsiaFluCap Simulator. [file 1471-2458-12-870-S5.pdf]

Additional file 5

Resource Model Parameters and Values

The AsiaFluCap Simulator

**Resource parameter**	**Description**	**Chosen value**	**Justification/notes (<u>all values</u> can be changed by users in the interface)**
p_{surge}	Proportion of total resource capacity that is available for influenza outbreak control and management.	0.12	Proportion based on [1-3].
$p_{threshold}$	Proportion of hospital beds that have to be occupied by cases before high pandemic activity period starts.	0.75	Arbitrary value. In our baseline scenarios we did not account for changes in resource use during the pandemic.
$p_{AbsenteeismPeak}$	Proportion of health care workers not available due to absenteeism.	0.15	10% absenteeism healthcare workers [4], 30% health care staff illness with work absences of up to 8 days [5], 40-70% absenteeism of health care staff [6].
$H_{hoursdayshiftsTotal}$	Total number of hours of both day shifts	16	We assumed a work day to be divided into two day shifts and one night.
$H_{hoursnightshift}$	Number of hours of night shift	8	We assumed a work day to be divided into two day shifts and one night.
$H_{workperday}$	Number of work hours per day	10	During the pandemic peak we assumed a 10 hour work day for health care workers.
$N_{workdaysweek}$	Number of work days per week	5	We assumed 5/7 days of work for health care workers during pandemic peak.
$H_{averageworkhoursperday}$	Average number of hours health care workers are available per day seen over one week.	7.1	$\mathbf{H}_{workday}*(\mathbf{N}_{workdaysperweek}/7)$
$\text{Þ}_{maskn95LOW}$ / $\text{Þ}_{maskn95HIGH}$	Number of masks N-95 / N-99 required per influenza case per day during low / high pandemic activity period.	1	10 N-95 respirators needed throughout hospital stay for non-ventilated hospitalised cases [7], mean 19.4 N95 masks per patients in the first 6 hours [8].
$F_{ventilatedmask95}$	Factor indicating the number of times more units are required per ventilated (ICU) case.	2	20 N-95 respirators needed throughout hospital stay for ventilated hospitalised cases [7]
$\text{Þ}_{SurgicalmaskLOW}$ / $\text{Þ}_{SurgicalmaskHIGH}$	Number of surgical masks required per influenza case per day during low / high pandemic activity period.	5	30 surgical masks needed per patient throughout hospital stay[7], 4,450 surgical masks needed (used by staff, patients and visitors) for 7 days for 29

			cases and WHO estimates are 2,436 [9],
$F_{ventilatedsurgmask}$	Factor indicating the number of times more units are required per ventilated (ICU) case.	$2^{1}/_{3}$	80 surgical masks needed per patient throughout hospital stay [7].
Þ$_{FaceshieldLOW}$ / Þ$_{FaceshieldHIGH}$	Number of face shields required per influenza case per day during low / high pandemic activity period.	1	Assumption based on [7, 9].
$F_{ventilatedfaceshield}$	Factor indicating the number of times more units are required per ventilated (ICU) case.	2	Assumption based on [7, 9].
Þ$_{GlovespairLOW}$ / Þ$_{GlovespairHIGH}$	Number of gloves (in pairs) required per influenza case per day during low / high pandemic activity period.	4	40 surgical gloves needed per patient throughout hospital stay [7], 8400 gloves (pairs) needed for 7 days for 29 cases and WHO estimates are 406 gloves (pairs) [9]. Mean 25.1 pairs of gloves per patient in the first 6 hours [8].
$F_{ventilatedgloves}$	Factor indicating the number of times more units are required per ventilated (ICU) case.	2½	100 surgical gloves needed per ventilated patient throughout hospital stay [7].
Þ$_{GownsLOW}$ / Þ$_{GownsHIGH}$	Number of coverall gowns required per influenza case per day during low / high pandemic activity period.	3	Assumption based on [9]. Mean 22.1 gowns per patients in first 6 hours [8].
$F_{ventilatedgowns}$	Factor indicating the number of times more units are required per ventilated (ICU) case.	2	Assumption based on [9].
p_{vac}	Proportion of (susceptible) population that will be vaccinated	-	Value set to 0 for baseline scenario. Users can include vaccination in interface (and indicate whether only risk groups are vaccinated or the whole population).
$N_{vaccinperperson}$	Number of vaccines per person	-	For instance: 1 or 2 doses needed per person.
v	Vaccine efficacy	-	Proportion which can be varied between 0 – 1.
Þ$_{antivirals}$	Courses (oseltamivir) per new influenza case (receiving antivirals)	1	One course defined as ten capsules (75 mg twice daily for five days) [10-14].

$Þ_{antibioticsLOW}$ / $Þ_{antibioticsHIGH}$	Antibiotics (grams) required per influenza case per day during low / high pandemic activity period.	0.420	Course is 500 mg twice daily for 5 days [15-17]. We assumed that cases only receive one course during their stay. An average hospital stay was assumed to be 12 days for non-ventilated cases: 5000 mg / 12 days.
$F_{ventilatedAntibiotics}$	Factor indicating the number of times more antibiotics are required per ventilated (ICU) case.	$^{12}/_{13}$	Course is 500 mg twice daily for 5 days [15-17]. We assumed that all cases only receive 1 course during their stay. As an average hospital stay was assumed to be 13 days for ventilated cases, we used: non-ventilated depletion rate times (12 days / 13 days).
$p_{NonventilatedAntibiotics}$	Proportion non-ventilated hospitalised cases requiring antibiotics.	0.74	74% of normal hospitalised cases required antibiotics [15].
$p_{VentilatedAntibiotics}$	Proportion ventilated hospitalised cases requiring antibiotics.	0.95	95% of ICU cases required antibiotics [15].
$Þ_{IVfluidsLOW}$ / $Þ_{IVfluidsHIGH}$	IV fluids (liters) required per influenza case per day during low / high pandemic activity period.	2.5	Assumption based on: hospital standard IV fluid regime 2.5 L/25 h) [18].
$F_{ventilatedIVfluids}$	Factor indicating the number of times more antibiotics are required per ventilated (ICU) case.	1	Assumption based on: hospital standard IV fluid regime 2.5 L/25 h) [18].
$p_{NonventilatedIVfluids}$	Proportion of non-ventilated cases receiving IV Fluids.	1	We assumed that all hospitalised cases received IV fluids (these proportions can be changed in the interface).
$p_{VentilatedIVfluids}$	Proportion of ventilated cases receiving IV Fluids.	1	
$R_{DayNonVentMDphysicians}$	Ratio medical doctors / physicians : non-ventilated hospitalised cases, day shift	1 : 10	One physician can take care for 10 cases during day shift [2, 7].

$R_{NightNonVentMDphysicians}$	Ratio medical doctors / physicians : non-ventilated hospitalised cases, night shift.	1 : 40	One physician can take care for 40 cases during night shift [2, 7]
$R_{DayVentMDphysicians}$	Ratio medical doctors/ physician : ventilated hospitalised cases, day shift.	1 : 4	Assumed based on [2, 7].
$R_{NightVentMDphysicians}$	Ratio medical doctors/ physicians : ventilated hospitalised cases, night shift.	1 : 4	Assumed based on [2, 7].
$p_{casesvisitingGP}$	Proportion of cases outside the hospital requiring a General Practitioners / Primary Care Physicians	0.08	Cumulative attack rate for GP consultants of 8.26% during H1N1 pandemic [19].
$H_{hoursGPneededpercase}$	Average duration of one consultation (in hours)	0.25	We assumed that one consultation takes 15 min (minutes/60). One mild case or outpatient was assumed to require one consultation during his/her days of illness.
$R_{DayNonVentInternalMS}$	Ratio internal medicine specialist: non-ventilated hospitalised cases, day shift	1 : 100	Assumptions for the AsiaFluCap model, based on [9]. Values can be changed in the interface.
$R_{NightNonVentInternalMS}$	Ratio internal medicine specialist: non-ventilated hospitalised cases, night shift	1 : 200	
$R_{DayVentInternalMS}$	Ratio internal medicine specialist : ventilated hospitalised cases, day shift	1 : 50	
$R_{NightVentInternalMS}$	Ratio internal medicine specialist : ventilated hospitalised cases, night shift	1 : 100	
$R_{DayNonVentOtherDoctors}$	Ratio other doctors (e.g. surgeons, pediatricians, obstetricians, etc.): non-ventilated hospitalised cases, day shift	1 : 15	Assumptions for the AsiaFluCap model, based on [9]. Values can be changed in the interface.

$R_{NightNonVentOtherDoctors}$	Ratio other doctors : non-ventilated hospitalised cases, night shift	1 : 40	
$R_{DayVentOtherDoctors}$	Ratio other doctors : ventilated hospitalised cases, day shift	1 : 10	
$R_{NightVentOtherDoctors}$	Ratio other doctors: ventilated hospitalised cases, night shift	1 : 20	
$R_{DayNonVentOtherNurses}$	Ratio nurses : non-ventilated hospitalised cases, day shift	1 : 5	Assumption based on [7, 9].
$R_{NightNonVentOtherNurses}$	Ratio nurses : non-ventilated hospitalised cases, night shift	1 : 10	Assumption based on [7, 9].
$R_{DayVentOtherNurses}$	Ratio nurses : ventilated hospitalised cases, day shift	1 : 1	Assumption based on [7, 9].
$R_{NightVentOtherNurses}$	Ratio nurses : ventilated hospitalised cases, night shift	1 : 2	Assumption based on [7, 9].
$H_{pharmacistpercase}$	Number of hours a pharmacists need per influenza case.	20/60	For preparing AV courses, and checking drug supplies in hospital (every morning and afternoon)[9]. We assumed that a pharmacist would require 20 minutes in total for one influenza case.
$R_{LaboratoryTCases}$	Ratio laboratory technicians: influenza cases.	1 : 30	This ratio implicates the number of cases which one laboratory technician can process (virus isolation, etc.) during one work day. Note that processing/testing clinical specimens takes > 1 day [20].
$R_{PublicHealthPCases}$	Ratio Public Health Personnel : influenza cases	1 : 50	This ratio implicates the number of cases one staff member can process during one work day.
$R_{VolunteersCases}$	Ratio volunteers : influenza cases	1 : 5	We assumed that volunteers can substitute nurses in case needed.
$R_{AdminStaffCases}$	Ratio administrative staff (hospital) : influenza cases.	1 : 25	We assumed that per certain number of cases one administrative staff member is required in the hospital (for instance per ward) [9].

$H_{AmbulanceCase}$	Total duration (in hours) of transport per influenza case.	2	Average mission duration per patient is two hours [21].
$p_{casesambulances}$	Proportion of all hospitalised cases that requires ambulance.	0.15	Around 10% of all hospitalised influenza cases required transport, to the hospital or to other advanced health care facilities [22, 23] , 83% of all hospitalised cases uses one [24].
$H_{OthertransportCase}$	Total duration (in hours) of transport per influenza case.	2	Average mission duration per patient is two hours [21].
$p_{casesOthertransport}$	Proportion of all hospitalised cases that requires other transport vehicles.	0.05	Assumption based on: around 10% of all hospitalised influenza cases required transport, to the hospital or to other advanced health care facilities [22, 23].
$H_{XrayCase}$	Total duration of one x-ray scan (in hours)	30 / 60	We assumed that one x-ray takes 30 minutes.
$p_{XrayCase}$	Proportion of all ventilated hospitalised cases undertaking an x-ray.	0.2	We assumed that only a proportion of all ventilated cases undertook an x-ray. Assumption based on: one patient required a portable chest x-ray [9], 50% of cases in nursing home residents had x-ray taken [25].

References

1. Kanter RK, Moran JR: **Hospital emergency surge capacity: an empiric New York statewide study**. *Ann Emerg Med* 2007, **50**(3):314-319.
2. Putthasri W, Lertiendumrong J, Chompook P, Tangcharoensathien V, Coker R: **Capacity of Thailand to contain an emerging influenza pandemic**. *Emerg Infect Dis* 2009, **15**(3):423-432.
3. Schull MJ, Stukel TA, Vermeulen MJ, Guttmann A, Zwarenstein M: **Surge capacity associated with restrictions on nonurgent hospital utilization and expected admissions during an influenza pandemic: lessons from the Toronto severe acute respiratory syndrome outbreak**. *Acad Emerg Med* 2006, **13**(11):1228-1231.
4. Nap RE, Andriessen MP, Meessen NE, Miranda Ddos R, van der Werf TS: **Pandemic influenza and excess intensive-care workload**. *Emerg Infect Dis* 2008, **14**(10):1518-1525.

5. Challen K, Bentley A, Bright J, Walter D: **Clinical review: mass casualty triage--pandemic influenza and critical care**. *Crit Care* 2007, **11**(2):212.
6. Anderson TA, Hart GK, Kainer MA: **Pandemic influenza-implications for critical care resources in Australia and New Zealand**. *Journal of critical care* 2003, **18**(3):173-180.
7. Krumkamp R, Kretzschmar M, Rudge JW, Ahmad A, Hanvoravongchai P, Westenhoefer J, Stein M, Putthasri W, Coker R: **Health service resource needs for pandemic influenza in developing countries: a linked transmission dynamics, interventions and resource demand model**. *Epidemiol Infect* 2011:1-9.
8. Swaminathan A, Martin R, Gamon S, Aboltins C, Athan E, Braitberg G, Catton MG, Cooley L, Dwyer DE, Edmonds D *et al*: **Personal protective equipment and antiviral drug use during hospitalization for suspected avian or pandemic influenza**. *Emerg Infect Dis* 2007, **13**(10):1541-1547.
9. Phin NF, Rylands AJ, Allan J, Edwards C, Enstone JE, Nguyen-Van-Tam JS: **Personal protective equipment in an influenza pandemic: a UK simulation exercise**. *J Hosp Infect* 2009, **71**(1):15-21.
10. Carrat F, Luong J, Lao H, Salle AV, Lajaunie C, Wackernagel H: **A 'small-world-like' model for comparing interventions aimed at preventing and controlling influenza pandemics**. *BMC Med* 2006, **4**:26.
11. Ciofi degli Atti ML, Merler S, Rizzo C, Ajelli M, Massari M, Manfredi P, Furlanello C, Scalia Tomba G, Iannelli M: **Mitigation measures for pandemic influenza in Italy: an individual based model considering different scenarios**. *PLoS One* 2008, **3**(3):e1790.
12. Germann TC, Kadau K, Longini IM, Jr., Macken CA: **Mitigation strategies for pandemic influenza in the United States**. *Proc Natl Acad Sci U S A* 2006, **103**(15):5935-5940.
13. Longini IM, Jr., Halloran ME, Nizam A, Yang Y: **Containing pandemic influenza with antiviral agents**. *Am J Epidemiol* 2004, **159**(7):623-633.
14. van Genugten ML, Heijnen ML, Jager JC: **Pandemic influenza and healthcare demand in the Netherlands: scenario analysis**. *Emerg Infect Dis* 2003, **9**(5):531-538.
15. Jain S, Kamimoto L, Bramley AM, Schmitz AM, Benoit SR, Louie J, Sugerman DE, Druckenmiller JK, Ritger KA, Chugh R *et al*: **Hospitalized patients with 2009 H1N1 influenza in the United States, April-June 2009**. *N Engl J Med* 2009, **361**(20):1935-1944.
16. World Health Organization (WHO): **Pocket book of hospital care for children Guidelines for the management of common illnesses with limited resources.** [http://www.who.int/child_adolescent_health/news/events/2011/14_02_2011/en/]
17. World Health Organization (WHO): **Reducing excess mortality from common illnesses**.[http://www.who.int/diseasecontrol_emergencies/common_illnesses2008_6.pdf]
18. Vermeulen H, Hofland J, Legemate DA, Ubbink DT: **Intravenous fluid restriction after major abdominal surgery: a randomized blinded clinical trial**. *Trials* 2009, **10**:50.

19. D'Ortenzio E, Renault P, Jaffar-Bandjee MC, Gauzere BA, Lagrange-Xelot M, Fouillet A, Poubeau P, Winer A, Bourde A, Staikowsky F *et al*: **A review of the dynamics and severity of the pandemic A(H1N1) influenza virus on Reunion island, 2009**. *Clin Microbiol Infect* 2010, **16**(4):309-316.
20. World Health Organization (WHO): **Manual for the laboratory diagnosis and virological surveillance of influenza**. [http://whqlibdoc.who.int/publications/2011/9789241548090_eng.pdf]
21. Ciapetti M, Cianchi G, Zagli G, Greco C, Pasquini A, Spina R, Batacchi S, Bonizzoli M, Bonacchi M, Lazzeri C *et al*: **Feasibility of inter-hospital transportation using extra-corporeal membrane oxygenation (ECMO) support of patients affected by severe swine-flu(H1N1)-related ARDS**. *Scand J Trauma Resusc Emerg Med* 2011, **19**:32.
22. D'Ancona G, Capitanio G, Chiaramonte G, Serretta R, Turrisi M, Pilato M, Arcadipane A: **Extracorporeal membrane oxygenator rescue and airborne transportation of patients with influenza A (H1N1) acute respiratory distress syndrome in a Mediterranean underserved area**. *Interact Cardiovasc Thorac Surg* 2011, **12**(6):935-937.
23. Maritz J, Maree L, Preiser W: **Pandemic influenza A (H1N1) 2009: the experience of the first six months**. *Clin Chem Lab Med* 2010, **48**(1):11-21.
24. Hak E, Meijboom MJ, Buskens E: **Modelling the health-economic impact of the next influenza pandemic in The Netherlands**. *Vaccine* 2006, **24**(44-46):6756-6760.
25. Drinka PJ, Krause P, Nest L, Dissing M, Gravenstein S, Goodman BM: **The effect of culture-positive influenza type A on resource use and adverse events in nursing home residents**. *J Am Geriatr Soc* 2002, **50**(8):1416-1420.
