# Supplementary figures and images for: Development of a resource modelling tool to support decision makers in pandemic influenza preparedness: The AsiaFluCap Simulator
Source: BMC Public Health. 2012 Oct 12;12:870. doi: 10.1186/1471-2458-12-870 (PMC3509032; doi:10.1186/1471-2458-12-870)

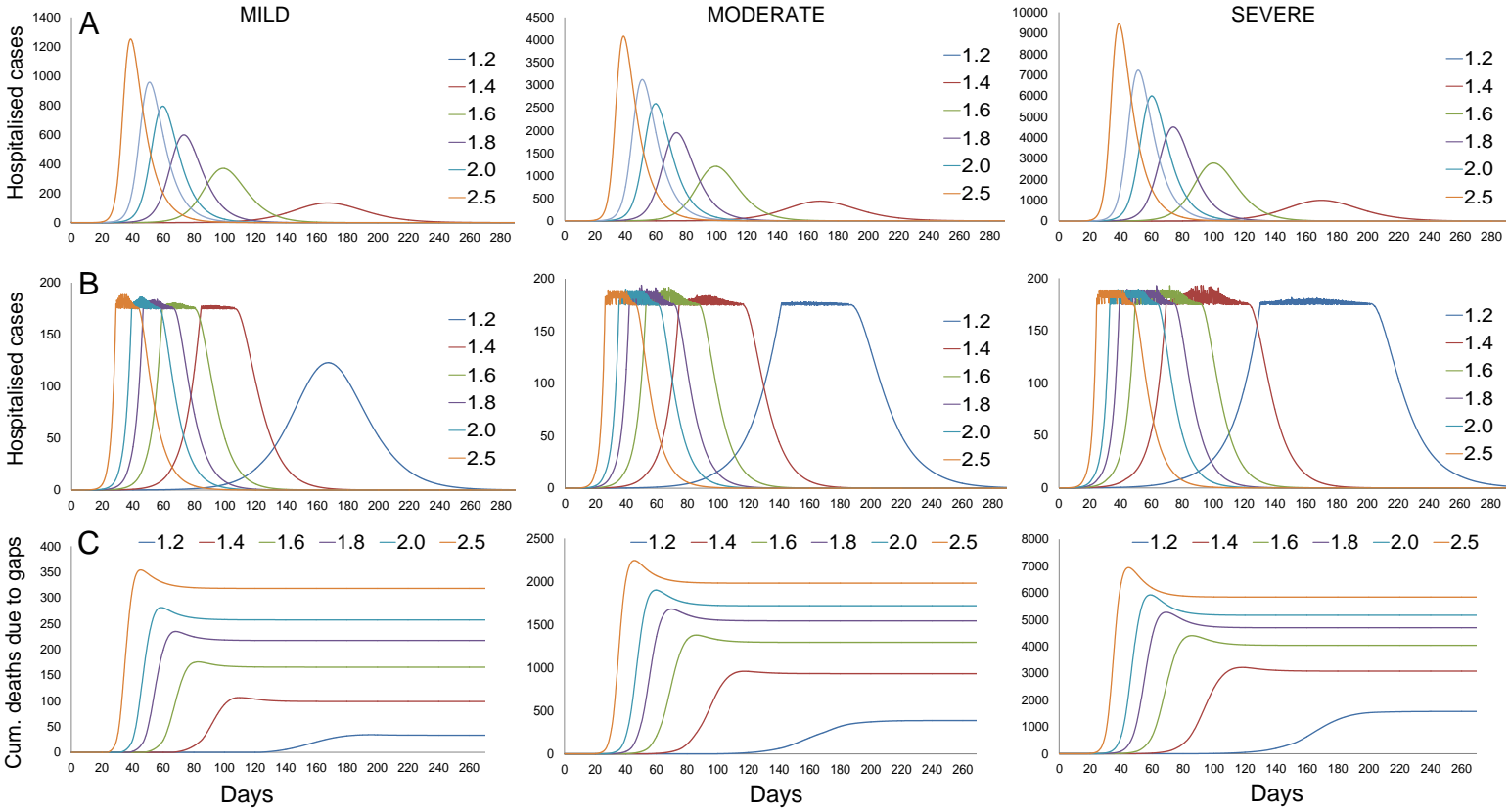

Supplement: Additional file 9 — File format: Adobe Acrobat Document (.pdf) Title: Sensitivity analysis performed with the AsiaFluCap Simulator. Description: Simulations were done for a region in Lao PDR (Vientiane Prefecture and Vientiane Province, n = 1,099,889) for a mild, moderate and severe pandemic scenario, and assuming different values for R0 (1.2, 1.4, 1.6, 1.8, 2.0 and 2.5) and no interventions (e.g. contact reduction, vaccination, etc.). A: Number of hospitalised cases when assuming sufficient hospital beds available in the region. B: Number of hospitalised cases when running simulations with actual available resources in the region. C: Cumulative number of deaths due to resource gaps (calculated by subtracting the number of deaths during scenarios simulated at A from the number of deaths estimated from the scenarios simulated at B). [file 1471-2458-12-870-S9.pdf]
